# HADHA ^K406^ Lactylation Regulates Metabolic Reprogramming and Suppresses Immune Responses in Gastric Cancer

**DOI:** 10.1002/advs.78291

**Published:** 2026-10-29

**Authors:** Maoxiao Feng, Qinlian Jiao, Jiankang Zhu, Yidan Ren, Xiaoyu Teng, Xin Su, Zigan Li, Yuxuan Cai, Xiaoyan Liu, Yunshan Wang, Guangyong Zhang

**Affiliations:** ^1^ Department of Clinical Laboratory Shandong Provincial Hospital Affiliated to Shandong First Medical University Jinan Shandong China; ^2^ Department of General Surgery The First Affiliated Hospital of Shandong First Medical University Jinan Shandong China; ^3^ Department of Clinical Laboratory Shandong Provincial Hospital Shandong University Jinan Shandong China; ^4^ Department of Clinical Laboratory Cheeloo College of Medicine The Second Hospital of Shandong University Shandong University Jinan Shandong China

**Keywords:** gastric cancer, HADHA ^K406^, HMGCR, lactylation modification

## Abstract

Gastric cancer (GC) represents a substantial global health challenge and is among the most prevalent and deadly malignancies worldwide. Emerging evidence suggests that protein lactylation plays a pivotal role in the progression of various cancers. However, the specific levels and regulatory mechanisms of protein lactylation in GC remain poorly understood. In this study, we detected elevated pan‐lactylation modification levels in GC tissues. Notably, lactylation at the K406 site of hydroxyacyl‐CoA dehydrogenase (HADHA) was significantly upregulated in GC tissues. This site‐specific lactylation modification enhanced GC progression. Disrupting lactylation at the HADHA ^K406^ site markedly reduced its enzymatic activity, thereby mitigating GC progression by impairing fatty acid β‐oxidation and glycolysis. Furthermore, inhibiting HADHA ^K406^ lactylation (HADHA K406la) suppressed the HMG‐CoA reductase (HMGCR)‐mediated cholesterol synthesis pathway, which, in turn, improved the immunosuppressive tumor microenvironment in GC. Finally, combining an HMGCR inhibitor with a lactate dehydrogenase A (LDHA) inhibitor effectively enhanced the immune response against GC and inhibited tumor progression. In conclusion, this study demonstrated that HADHA K406la regulates GC progression through its impact on metabolic pathways, suggesting its potential as a therapeutic target for GC treatment.

## Graphical abstract

HADHA K406 lactylation fuels fatty acid β‐oxidation and HMGCR‐dependent cholesterol biosynthesis, fostering immunosuppressive MDSCs and exhausted CD8^+^ T cells in gastric cancer. Loss of this modification blocks cholesterol production, remodels the tumor microenvironment, and tumor growth is suppressed via combined inhibition of HMGCR and LDHA.

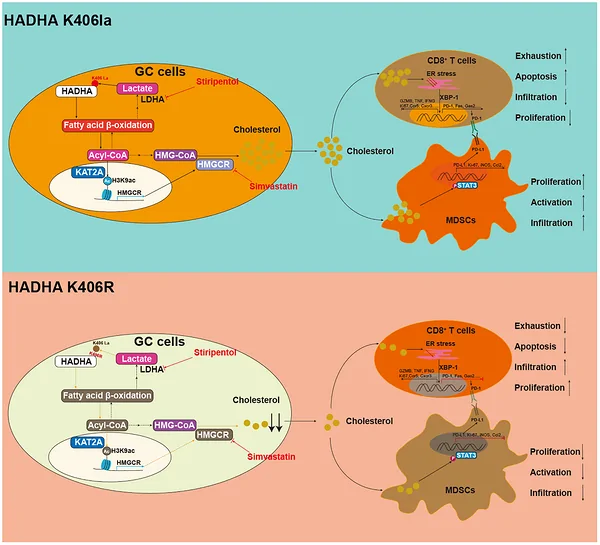

## 1 Introduction

Gastric cancer (GC) ranks among the five most frequently diagnosed malignancies and is the third leading cause of cancer‐related mortality worldwide [1]. GC exhibits marked molecular and phenotypic heterogeneity [2]. Established risk factors for GC include chronic *Helicobacter pylori* infection, older age, excessive dietary salt, and insufficient intake of fruits and vegetables [3, 4, 5]. The current treatment of patients with GC is suboptimal, as patients are often treated uniformly regardless of the disease subtype [6]. With in‐depth exploration of the genome and epigenome of GC using high‐throughput sequencing techniques, genetic and epigenetic factors such as gene mutations, chromosomal aberrations, differential gene expression, and epigenetic changes have been shown to play a role in the pathogenesis of GC [7, 8, 9]. Furthermore, as the immunogenomics of GC are better understood, novel immune checkpoint inhibitors may become increasingly important in GC treatment [10, 11]. The joint exploration of GC genomes, epigenetics, and immunogenomics will offer valuable tools for identifying predictive biomarkers and enabling personalized treatment.

Various types of posttranslational modifications (PTMs) have been shown to regulate various biological processes in the tumor microenvironment, including malignant cell proliferation [12, 13], immune cell activation [14], and metabolic reprogramming [15], suggesting that targeting PTMs has therapeutic potential in cancer. Recent studies have identified lysine lactylation (Kla) as a lactate‐derived posttranslational modification [16]. Kla was first characterized on core histones in a p300‐dependent manner [17] and was shown to modulate epigenetic programs in macrophages, promoting M2‐like gene expression in response to bacterial stimulation and thereby contributing to immune homeostasis [16]. Importantly, the abundance of intracellular lactate has been reported to correlate positively with Kla levels [16]. Studies have shown that Kla promotes HCC progression by regulating cellular metabolic processes [17]. Furthermore, METTL16 lactylation in GC promotes cuproptosis through m6A modification of FDX1 mRNA [18]. However, the mechanism by which nonhistone lactylation in GC regulates tumor progression remains unclear.

HADHA, also known as the α subunit of mitochondrial trifunctional protein (TFP), plays an important role in the final step of mitochondrial β‐oxidation of long‐chain fatty acids. Four α subunits (HADHA) and four β subunits (HADHB) comprise the mitochondrial TFP. HADHA has long‐chain 3‐hydroxyacyl‐CoA dehydrogenase (LCHAD) and enoyl‐CoA hydratase (ECH) activities, whereas HADHB functions as the 3‐ketoacyl‐CoA thiolase (KACT) component of the mitochondrial trifunctional protein [19]. Pathogenic variants in HADHA, or reductions in its enzymatic activity to less than approximately half of normal, are associated with severe LCHAD deficiency, characterized by hypoglycaemia, cardiomyopathy, and sudden death [20]. More recently, HADHA has emerged as an important lipid metabolic enzyme involved in tumorigenesis and progression in multiple malignancies, including hepatocellular carcinoma [21, 22], ovarian cancer [23], and malignant lymphoma [24]. Despite these findings in other cancer types, understanding of the role of HADHA lactate modification and its lactate modification sites in GC progression is currently limited.

In this study, a proteomic analysis of protein lactylation modifications in GC tissues and adjacent tissues revealed that the lactylation level of HADHA at the K406 site in GC tissues significantly increased and that its lactylation level promoted the progression of GC. We subsequently reported that the regulation of histone acetylation levels by HADHA ^K406^ lactylation (HADHA K406la) promoted the expression of HMGCR, which in turn regulated the synthesis of cholesterol in GC cells, resulting in an immunosuppressive GC microenvironment. In summary, we clarified the role of the HADHA K406la modification in the progression of GC, laying the foundation for its clinical application in the precise diagnosis and treatment of GC.

## 2 Materials and Methods

### 2.1 Human GC Samples

Histologically confirmed GC tissues and matched adjacent nontumorous samples used for lactylome and proteome analyses were collected at Shandong Provincial Qianfoshan Hospital. This retrospective observational study did not include any individually identifiable patient data. Written informed consent for publication was waived by the institutional research ethics review board of Shandong Provincial Qianfoshan Hospital. The proteomic study protocol was approved by the institutional research ethics review board (Approval No.: YXLL‐LY‐2022(068)). Resected specimens were snap‐frozen in liquid nitrogen and stored at –80°C until analysis. The clinicopathological features of the GC cohort are listed in Table S1. To evaluate the prognostic impact of pan‐lactylation in GC, we also employed a commercial GC tissue microarray (Super Biotek, Cat. No. STC1601; Figures 1H, and 2J). Clinicopathological information of patients on GC tissue microarrays, as well as grouping data of pan‐lactylation and HADHA K406la levels, are presented in Table S2.

**FIGURE 1:**
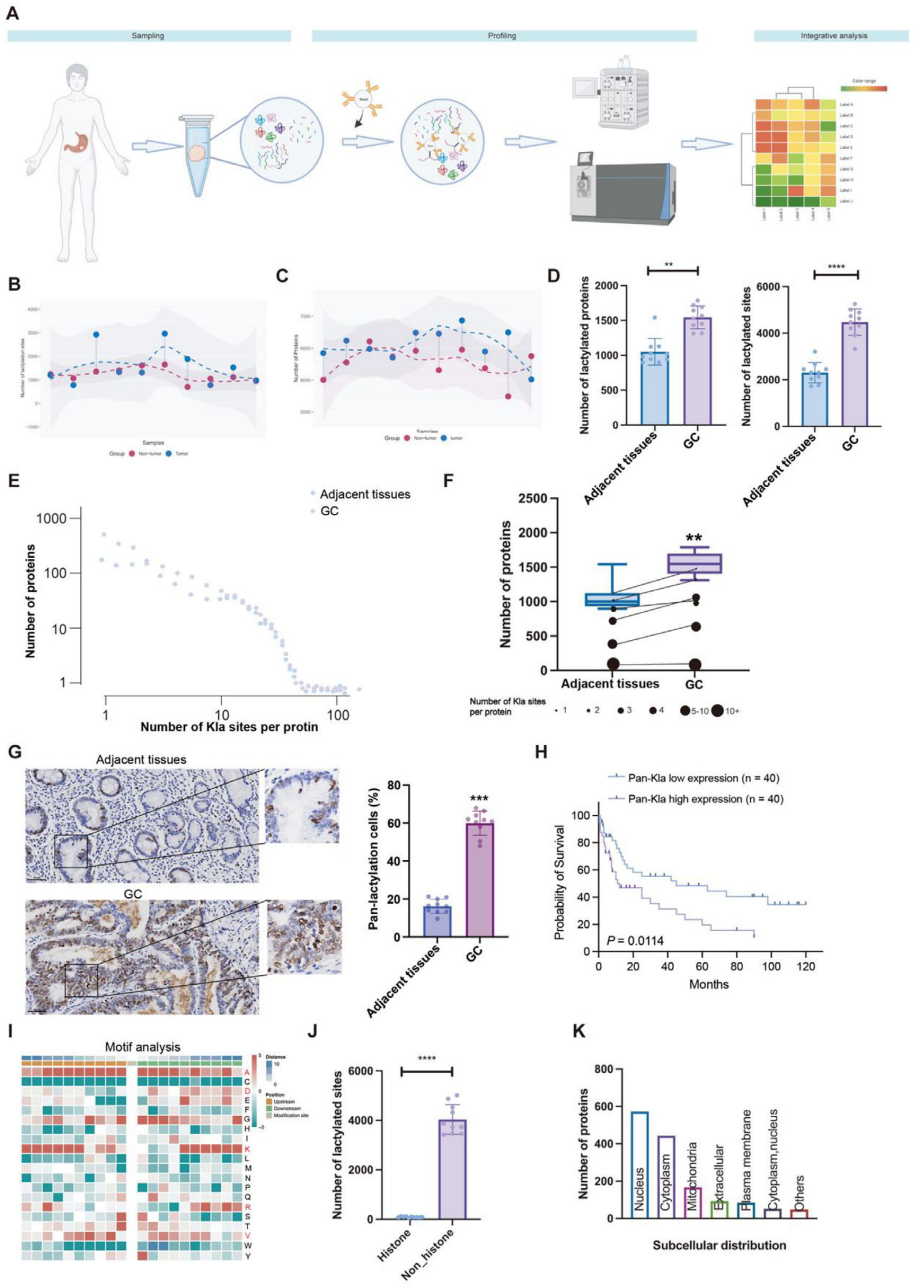
Characteristics and prognostic significance of protein lysine lactylation in gastric tumors. (A) Schematic of the lactylome and proteome analyses of human gastric tumor tissues and adjacent tissues by label‐free quantitation. (B) Overview of the Kla identifications. The dashed curves fitted by LASSO regression show the distribution of Kla in tumor (red, n = 10) and nontumor (blue, n = 10) samples. (C) Overview of the protein. The dashed curves fitted by LASSO regression show the distribution of Kla in tumor (red, n = 10) and nontumor (blue, n = 10) samples. (D) Box plots of Kla site identification in gastric tumor samples. (E, F) Numbers of Kla sites per protein in gastric tumor and adjacent tissues. (G) Immunohistochemical staining showing the pan‐Kla level in gastric tumor and adjacent tissues. The graph shows the results of the quantitative analysis of the pan‐lactylation level. Scale bars denote 50 µm. (H) Kaplan–Meier survival curve of overall survival for pan‐Kla density (pan‐Kla high, n = 40; pan‐Kla, n = 40) in GC patients. (I) Heatmap of the 21 amino acid compositions of the Kla site showing the frequency of the different amino acids in specific positions flanking the lactylated lysine. (J) Number of detected Kla sites on nonhistone and histone proteins. (K) Subcellular location of all the identified lactylated proteins.

**FIGURE 2:**
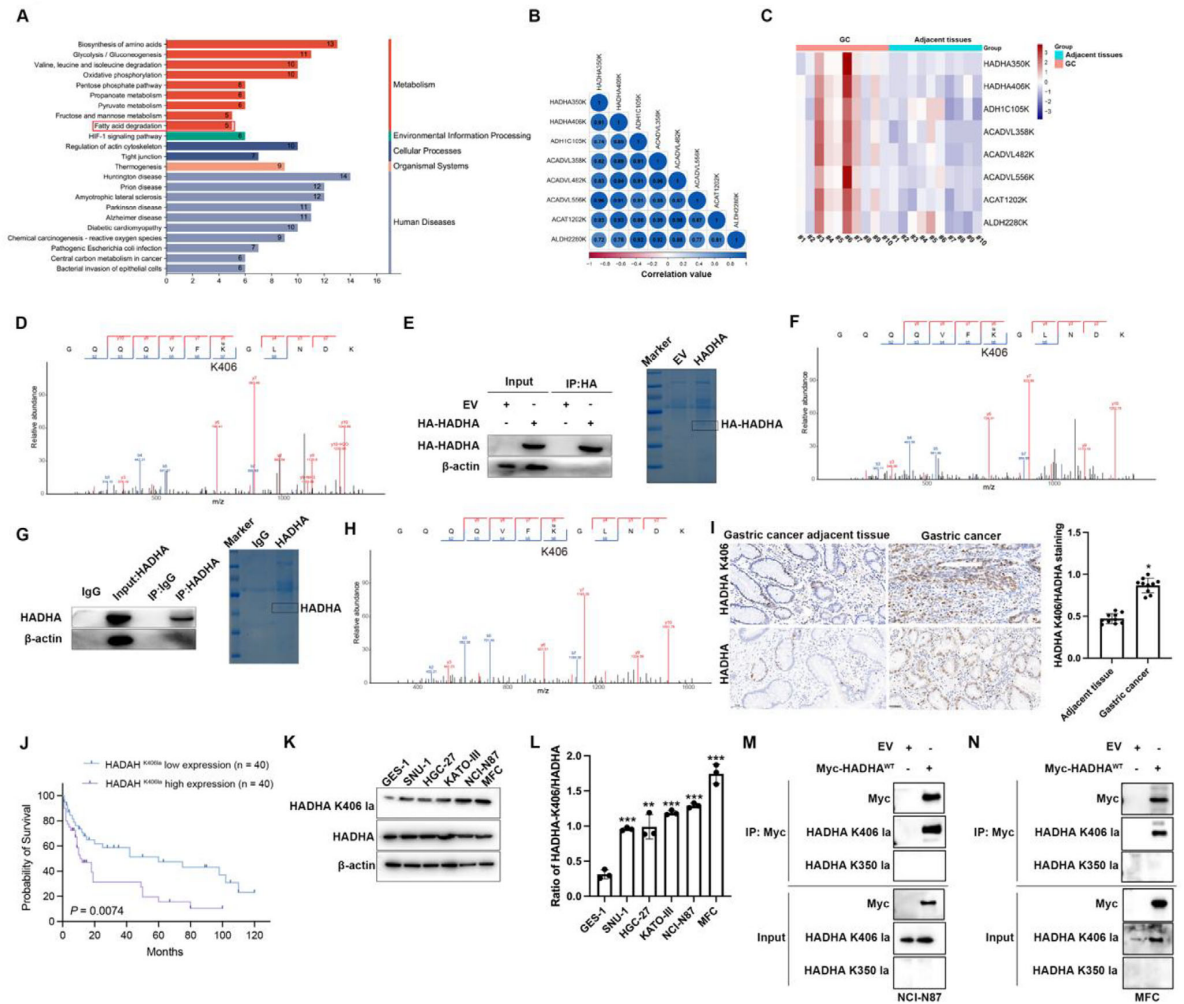
HADHA K406la is elevated in GC cells. (A) Pathway enrichment analysis was performed on the basis of the differentially expressed proteins (DEPs) whose FDR‐adjusted p value was less than 0.05 between GC and normal adjacent tissues. (B) A correlation analysis was conducted for proteins enriched in fatty acid degradation. (C) A heatmap was generated to visualize the expression levels of enzymes identified through GC lactylation analysis. (D) Representative spectrum for peptide‐containing HADHA K406la. (E–H) LC–MS/MS analysis of the lactylation sites of exogenous HA‐HADHA (E, F) and endogenous HADHA (G, H) in NCI‐N87 cells. (I) Immunohistochemical (IHC) staining showing the HADHA K406 and HADHA expression levels in GC and adjacent normal tissues. The graph shows the results of the quantitative analysis of HADHA K406/HADHA (mean ± SD, n = 10, scale bars: 50 µm). (J) Kaplan–Meier curve of overall survival for HADAH K406la density (HADHA K406la high, n = 40; HADHA K406la, n = 40) in GC patients. (K, L) The expression of total and lactylated HADHA at K406 in GC cells (KATO‐III, SNU‐1, NCI‐N87, HGC‐27 and MFC) and corresponding normal cells (GES‐1) was determined by western blotting using anti‐HADHA and anti‐HADHA‐K406 antibodies (mean ± SD, n = 3). (M, N) NCI‐N87 and MFC cells overexpressing Myc‐HADHA were subjected to immunoprecipitation with an anti‐Myc antibody, followed by western blotting using anti‐HADHA K406la or anti‐HADHA K350la antibodies to assess lactylation at specific HADHA sites. **p < 0.01 and ***p < 0.001.

### 2.2 Proteomics and Modified Proteomics

The sample was ground with liquid nitrogen into a cell powder and then transferred to a 5 mL centrifuge tube. Four volumes of lysis buffer (8 M urea and 1% protease inhibitor cocktail) were added to the cell powder, followed by sonication three times on ice using a high‐intensity ultrasonic processor (Scientz). For the PTM experiments, the following inhibitors were added to the lysis buffer: 3 µM trichostatin A (Merck, 58880‐19‐6), 50 mM NAM (Merck, BP460) for acetylation, and 1% phosphatase inhibitor (Merck, P0044) for phosphorylation. The remaining debris was removed by centrifugation at 12 000 g and 4°C for 10 min. Finally, the supernatant was collected, and the protein concentration was determined using a BeyoBCA kit (Beyotime, P0398L) according to the manufacturer's instructions.

### 2.3 LC‒MS/MS‐Based Peptide Analysis

Protein samples were reduced with 5 mM dithiothreitol at 56°C for 30 min and then alkylated with 11 mM iodoacetamide (Sigma–Aldrich, I6125) for 15 min in the dark. They were then diluted to < 2 M urea (Sigma–Aldrich, U5378) with 100 mM TEAB (Sigma–Aldrich, 241059). Digestion was performed at 37°C overnight at a trypsin (Merck, T2600000) to protein ratio of 1:50 and then at 1:100 for 4 h. Eluted peptides were purified using C18 solid‐phase extraction columns and freeze‐dried using a SpeedVac device to obtain lyophilized peptides. The peptides were dissolved in solvent A [0.1% formic acid (Sigma–Aldrich, 695076) and 2% acetonitrile (Sigma–Aldrich, 360457) in water] and directly loaded onto a homemade reversed‐phase analytical column (25 cm length, 100 µm i.d.) for both lactylome and proteomic analyses. The enriched Kla‐peptides were separated using a gradient of solvent B (0.1% formic acid in acetonitrile) from 7% to 24% over 42 min, from 24% to 32% over 12 min, and increased to 80% over 3 min, after which they were held at 80% for the last 3 min, all at a constant flow rate of 450 nL/min on a nanoElute ultra‐high‐resolution liquid chromatography (UHPLC) system (Bruker Daltonics). For proteomic analysis, the tryptic peptides were separated using a gradient of solvent B (0.1% formic acid in acetonitrile) from 6% to 24% over 70 min, 24% to 35% over 14 min, and increased to 80% over 3 min, after which they were held at 80% for the last 3 min, all at a constant flow rate of 450 nL/min on a nanoElute UHPLC system. All eluted peptides were subjected to a capillary source, followed by timsTOF Pro (Bruker Daltonics) mass spectrometry. Precursors and fragments were analyzed using a TOF detector, with an MS/MS scan range from 100 to 1700 m/z. The timsTOF Pro was operated in parallel accumulation fragmentation (PASEF) mode. Precursors with charge states of 0–5 were selected for fragmentation, and ten PASEF MS/MS scans were acquired per cycle. The dynamic exclusion was set at 30 s.

### 2.4 Database Search

The acquired MS and MS/MS data were searched using MaxQuant (version 1.6.15.0) in conjunction with the human SwissProt database and the reverse decoy database. The proteolytic enzyme trypsin was used, and two cleavage sites were missed. The precursor ion tolerance and peptide tolerance for the main search were set to 20 and 5 ppm, respectively. For the analysis of Kla‐modified peptide enrichment data, the variable modification set included lysine lactylation. The false discovery rate (FDR) was set to < 1% for peptide spectrum matching and site and protein identification. The expression of specific proteins in the samples was obtained from the MaxQuant result files. The protein levels of all the samples were analyzed using the MaxLFQ algorithm in MaxQuant software. Kla peptide levels were calculated on the basis of the raw spectral intensities and normalized to protein abundance.

### 2.5 HADHA Enzyme Activity Assay

Mitochondrial extracts were prepared by differential centrifugation of lysates from GC cells stably overexpressing either HADHA ^WT^ or HADHA ^K406R^. For the enzymatic assay, 10 µL of mitochondrial extract was added to 160 µL of reaction buffer (50 mM Tris‐HCl, pH 8.0; 0.1 mM EDTA), followed by the addition of 20 µL of NAD^+^ (10 mM) and 10 µL of acetoacetyl‐CoA (2 mM) as the substrate. The mixture was incubated at 37°C to initiate the reaction. At defined time points, 20 µL aliquots were collected and immediately quenched by the addition of 20 µL of ice‐cold 1 M HCl. All the samples were then transferred to a 96‐well plate, and the absorbance at 340 nm was recorded continuously for 5 min using a kinetic microplate spectrophotometer (BioTek), reflecting the reduction of NAD^+^ to NADH. A standard curve was generated using known concentrations of NADH, and HADHA enzymatic activity was calculated on the basis of the rate of change in the absorbance (ΔA340), as described previously [25].

### 2.6 Filipin III Staining

Cells were seeded onto glass coverslips in 24‐well plates and cultured for 48 h. After incubation, the cells were washed twice with phosphate‐buffered saline (PBS) and fixed with 4% paraformaldehyde (PFA) for 15 min at room temperature. To reduce autofluorescence, the cells were then incubated with 1.5 mg/mL glycine in PBS for 10 min. Following glycine treatment, the cells were permeabilized with 0.1% Triton X‐100 in PBS for 5 min at room temperature. After three washes with PBS, the cells were stained with Filipin III (50 µg/mL in PBS; Sigma–Aldrich, SAE0087) in the dark at room temperature for 1 h. The stained coverslips were then washed three times with PBS and mounted onto glass slides. Fluorescence images were acquired using a fluorescence microscope equipped with a DAPI (Solarbio, C0065) filter. Image analysis was performed using ImageJ software.

### 2.7 Cholesterol Quantification

Mouse tumor tissue was rinsed with cold PBS to remove residual blood. The tissue was then cut into small pieces and kept on ice. Homogenization was performed in 100 µL of detection buffer using a tissue grinder, followed by centrifugation to collect the supernatant. For cholesterol quantification, 50 µL of the total cholesterol reaction mixture was added to each cholesterol sample according to the manufacturer's instructions (Cholesterol Quantification Kit, Abcam, ab65390). The mixture was thoroughly mixed and incubated at 37°C in the dark for 60 min. The absorbance was measured at 570 nm using a microplate reader. A standard curve was generated, and the cholesterol content in the samples was calculated on the basis of the standard curve.

### 2.8 Single‐Cell RNA Sequencing (scRNA‐Seq)

Tumor tissues were harvested and minced into small fragments using sterile scissors, followed by enzymatic dissociation in a digestion cocktail containing collagenase IV (0.5 mg/mL, Sigma–Aldrich, C5138) and DNase I (50 U/mL, Sigma–Aldrich, DN25). The digestion was carried out at 37°C for 20–30 min with intermittent gentle mixing. The resulting cell suspension was passed through a 40‐µm cell strainer to remove debris, and red blood cells were lysed using RBC lysis buffer. The cells were then washed with PBS and centrifuged at 300 × *g* for 5 min at 4°C. The pellet was resuspended in PBS containing 0.04% BSA (Sigma–Aldrich, V900933), and cell viability was assessed using trypan blue staining under a light microscope. Only samples with >85% viable cells were processed further. Single‐cell capture, barcoded cDNA synthesis, and library preparation were performed using the Chromium Next GEM Single‐Cell 3’ v3.1 Kit (10x Genomics, CG1000268) following the manufacturer's protocol. The prepared libraries were sequenced on an Illumina NovaSeq 6000 using a paired‐end 150 bp (PE150) read configuration, targeting approximately 50 000 reads per cell. The raw sequencing data were demultiplexed and converted into FASTQ format using Cell Ranger (10x Genomics, v6.0). Reads were aligned to the mm10 (mouse) reference genome using STAR, and gene expression matrices were generated. For the HADHA ^WT^ group, the median UMI count per cell was 6038, the median number of genes per cell was 2035, and the median percentage of mitochondrial genes was 2.72%. In the HADHA ^K406R^ group, the median UMI count per cell was 5541, the median number of genes per cell was 1915, and the median percentage of mitochondrial genes was 2.57%. Comparable quality metrics were observed between the two groups, suggesting no obvious batch effect or quality bias.

### 2.9 Custom Antibody Generation

Polyclonal antibodies targeting HADHA K406la and HADHA K350la were generated in rabbits. The specific immunopeptide sequences ultimately used for immunization were as follows: for HADHA K406la, ALDRGQQQVFK(la)GLNDKVK and for HADHA K350la, GLYHGQVLCK(la)KNKFGA. Corresponding nonlactylated peptides were also synthesized as controls. All synthetic immunogens were conjugated to keyhole limpet haemocyanin (KLH) using standard chemical conjugation methods. Rabbits were immunized subcutaneously according to a standard immunization protocol, including primary immunization and multiple booster injections. After the immunization procedure was complete, antisera were collected and subjected to subsequent affinity purification using columns covalently coupled with the corresponding lactylated peptides. Briefly, synthesized lactylated peptides were covalently conjugated to NHS‐activated Sepharose 4B resin in coupling buffer containing 0.2 M NaHCO_3_ and 0.5 M NaCl (pH 8.3) and incubated overnight at 4°C under gentle rotation. The residual active sites on the resin were blocked with 0.1 M Tris‐HCl buffer (pH 8.0) for 2 h at room temperature, and the resin was alternately washed with acidic buffer (0.1 M acetic acid and 0.5 M NaCl; pH 4.0) and basic buffer (0.1 M Tris‐HCl and 0.5 M NaCl; pH 8.0) to thoroughly remove unbound peptides and impurities. The collected crude antisera were diluted with binding buffer consisting of 20 mM Tris‐HCl and 150 mM NaCl (pH 7.4) and slowly loaded onto the prepared peptide affinity column. After sample loading, the column was extensively washed with the same binding buffer to completely remove unbound and nonspecifically adsorbed impurities. The target‐specific antibodies were eluted using elution buffer containing 0.1 M glycine‐HCl (pH 2.7), and each eluted fraction was immediately neutralized with 1 M Tris‐HCl (pH 8.5) to maintain antibody activity. All neutralized antibody fractions were pooled and concentrated using 30 kDa molecular weight cut‐off ultrafiltration tubes, followed by buffer exchange into storage buffer containing 20 mM Tris‐HCl, 150 mM NaCl and 50% glycerol (pH 7.4). The final purified antibodies were aliquoted and stored at −80°C for long‐term preservation with strict avoidance of repeated freeze‒thaw cycles. The specificity of the purified antibodies was validated by dot blot analysis, which confirmed the selective recognition of the target lactylated epitopes. The entire antibody generation and purification process was performed by Jingjie Biotechnology Co., Ltd.

### 2.10 Dot Blot Assay

To assess the specificity of the self‐developed HADHA K406la antibody, a dot blot assay was performed using synthetic peptides. Three peptides corresponding to the HADHA K406 site were synthesized: (1) unmodified K406 peptide, (2) K406 lactylated peptide (HADHA‐K406la La‐1: QQQVFK(la)GLNDKVK, HADHA‐K406la La‐2: DRGQQQVFK(la)GLNDKVK, HADHA‐K406la La‐3: ALDRGQQQVFK(la)GLNDKVK, HADHA‐K406la Non‐4: RGQQQVFKGLNDKVK), and (3) K406 acetylated peptide (K406ac, DRGQQQVFK(ac)GLNDKVK) and K350 lactylated peptide (HADHA‐K350la La1: GQVLCK(la)KNKFGA, HADHA‐K350la La2: HGQVLCK(la)KNKFGA, K350la La3: GLYHGQVLCK(la)KNKFGA, HADHA‐K350la Non‐4: HGQVLCKKNKFGA) (GL Biochem, Shanghai, China). Each peptide was dissolved in PBS at a concentration of 1 µg/µL. Serial dilutions (1 µg, 200 ng, and 40 ng per spot) were dotted onto a dry polyvinylidene difluoride (PVDF) membrane (Millipore), followed by air‐drying for 30 min at room temperature. The membrane was then blocked with 5% bovine serum albumin (BSA) in Tris‐buffered saline with 0.1% Tween‐20 (TBST) for 1 h at room temperature and incubated overnight at 4°C with the HADHA K406la antibody (1:1000 dilution in TBST containing 1% BSA). After three washes with TBST, the membrane was incubated with a horseradish peroxidase (HRP)‐conjugated goat anti‐rabbit IgG secondary antibody (1:5000; Cell Signaling Technology) for 1 h at room temperature. The signal was detected using enhanced chemiluminescence (ECL) reagent (Thermo Fisher Scientific) and imaged using a ChemiDoc XRS+ system (Bio‐Rad).

### 2.11 Immunohistochemistry

Fresh tissue samples were fixed in neutral‐buffered 10% formalin for 24 h, dehydrated in 70% ethanol, embedded in paraffin, quickly embedded in Tissue‐Tek OCT compound (Sakura, 4583), and snap‐frozen for frozen block preparation. Lactate expression of HADHA K406la was detected by immunohistochemical staining with specific antibodies (HADHA K406la antibody was ordered from Jingjie Biotechnology Co., Ltd., 1:1000). The proportion of positively stained tumor cells and staining intensity were scored by two independent observers.

### 2.12 Molecular Simulation Docking

The amino acid sequence of HADHA was obtained from NCBI, and the protein structure of HADHA was simulated using AlphaFold 2 software. PyMOL was used to add a lactylation modification to the lysine residue at position 406 of the HADHA protein. Molecular dynamics simulations of the modified protein structure were performed using Gromacs software. The simulation process includes energy minimization, the NVT ensemble, and the NPT ensemble.

### 2.13 Cell Lines and Cell Culture

GES‐1, NCI‐N87, SNU‐1, HGC‐27, KATO‐III, and MFC cells were obtained from the American Type Culture Collection (ATCC). Cells were grown in high‐glucose Dulbecco's modified Eagle medium (DMEM; Biological Industries) supplemented with 10% foetal bovine serum (FBS; Biological Industries). All the cultures were maintained at 37°C and 5% CO_2_.

### 2.14 MTT Assay

Cells were seeded in 96‐well plates at a density of 5 × 10^3^ cells per well in a total volume of 200 µL per well. After 24, 48, and 72 h of incubation, 20 µL of MTT solution (5 mg/mL, Solarbio, IM0280) was added to each well. The cells were then incubated for an additional 4 h at 37°C. Following incubation, the medium was removed, and 100 µL of dimethyl sulfoxide (DMSO) was added to each well to dissolve the formazan crystals. The plate was gently shaken for 10 min to ensure complete dissolution. The absorbance was measured at 570 nm using a microplate reader.

### 2.15 Transwell and Migration Assays

For the Transwell assay (3422; Corning, NY, USA), 2 × 10^5^ cells were suspended in 100 µL of FBS‐free RPMI 1640 medium, seeded into the upper chambers, and placed in a 24‐well plate containing 600 µL of RPMI 1640 medium supplemented with 10% FBS in the lower chambers. After being incubated at 37°C, the cells at the bottom of the membrane were fixed with methanol and stained with crystal violet. The number of cells in five randomly selected microscopic fields was counted.

### 2.16 Mice and GC Mouse Model

C57BL/6J mice and nude mice were purchased from the Model Animal Research Center of Nanjing University. C57BL/6J and nude mice were subcutaneously inoculated with MFC or NCI‐N87 cells (1 × 10^6^). MFC cells were originally established in 615 mice, and allograft transplantation into C57BL/6J mice may cause mild immune rejection, which was consistent across all the experimental groups. The tumor size was calculated as length × (width^2^)/2. Survival was recorded daily. Tumor‐bearing mice were euthanized when the tumor size exceeded 2000 mm^3^. All the animals were housed and maintained under specific pathogen‐free conditions. The animal protocols were approved by the Shandong University Animal Care and Use Committee (ID: KYLL‐2023‐006).

### 2.17 Isotope Labeling Experiments With [2,4‐13C]‐Citrate

To investigate the positive feedback mechanism between lactate levels and HADHA lactylation modification, we performed an isotope tracing experiment using [2,4‐13C]‐labeled citrate (Cambridge Isotope Laboratories, Inc.). Briefly, GC cells were cultured in Dulbecco's modified Eagle medium (DMEM) supplemented with 10% foetal bovine serum under standard conditions (37°C, 5% CO_2_). Upon reaching 70%–80% confluence, the cells were washed with PBS and switched to custom medium supplemented with 4 mM [2, 4‐13C]‐citrate as the sole carbon source for 24 h. After incubation, the cells were harvested, and the metabolites were extracted using a methanol:water (80:20, v/v) solution. Lactate and HADHA K406la levels were quantified via liquid chromatography‒mass spectrometry (LC‒MS/MS).

### 2.18 Flow Cytometry

MagniSort Positive Selection Beads (eBioscience) were used to isolate CD8^+^ T cells following the manufacturer's instructions. Cells were stimulated with phorbol 12‐myristate 13‐acetate (PMA; 100 ng/mL) and ionomycin (1 µM) for 6 h and collected for flow cytometry analysis. Flow cytometry was performed using a FACS Aria Sorp system (BD FAC Aria II Sorp System). FlowJo 10.4 software was used to analyze the data. Flow cytometry antibody information is shown in Table S3.

### 2.19 ChIP‐Seq

The cells were cross‐linked with 1% fresh formaldehyde for 10 min at room temperature, neutralized with glycine for 5 min, and lysed in SDS lysis buffer. The cross‐linked DNA was then sheared into fragments of 200–1000 bp in length using UCD‐300 (Bioruptor). ChIP was performed using a Chromatin Immunoprecipitation Kit according to the manufacturer's instructions (Millipore, 17–371) to obtain ChIP‐enriched DNA. The 75‐nt sequence reads generated by Illumina sequencing were mapped to the genome using the Burrows–Wheeler Aligner algorithm with default settings. Only reads that passed the Illumina purity filter, aligned with no more than two mismatches, and were mapped uniquely to the genome were used in subsequent analysis. Peak calling was performed using MACS (version 2.1.0). The average peak values of all active regions in the gene and within the gene margin were used to calculate the differentially enriched genes.

### 2.20 RNA‐Seq

mRNA was purified from the total RNA from HADHA ^WT^ or HADHA ^K406R^ SGC cells for RNA‐seq. RNA‐seq libraries were generated using the NEBNext Ultra RNA Library Prep Kit for Illumina (NEB) and sequenced on an Illumina platform with 125 bp/150 bp paired‐end reads. Clean data were obtained by removing reads containing adapters, poly‐N, and low‐quality reads from the raw data. Clean reads were mapped to the reference genome HISAT2 (version 2.0.5) on the basis of the gene model annotation file. Feature Counts v1.5.0‐p3 was used to count the read numbers mapped to each gene. Fragments per kilobase per million mapped reads for each gene were calculated on the basis of the length of the gene and the read count mapped to this gene. Differential gene expression analyses were performed using the DESeq2 package.

### 2.21 Measurement of Intracellular Acetyl‐CoA Levels

The GC cell lines were washed twice with PBS and lysed with lysis buffer.

The lysate was sonicated and centrifuged at 8000 × *g* for 10 min at 4°C, after which the cell mixture was resuspended. The intracellular acetyl‐CoA content was determined using an acetyl‐CoA content assay kit (Boxbio, Beijing, China) according to the manufacturer's instructions.

### 2.22 ChIP‒qPCR

The ChIP assay was performed using an EZ‐Magna ChIP Chromatin Immunoprecipitation Kit (Abcam, UK) according to the manufacturer's instructions. Briefly, we fixed 5 × 10^6^ HADHA^WT^ or HADHA^K406R^ SGC cells in 1% formaldehyde for 10 min at ambient temperature. The fixed cells were harvested, lysed, and sonicated. Antibodies against H3K9ac and rabbit IgG were used for immunoprecipitation (IP), and PCR amplification of the precipitated DNA was performed. The primer information is provided in Table S4.

### 2.23 Western Blotting

The cells were subsequently washed in ice‐cold PBS and solubilized in lysis buffer (25 mM Tris‐HCl (pH 7.6), 200 mM NaCl, 1 mM EDTA, 1% Igepal CA‐630, 0.1% Na‐deoxycholate, and 0.1% SDS supplemented with a mixture of protease inhibitors). The lysates were incubated on ice for 30 min and centrifuged at 12 000 rpm for 25 min. All the supernatants were collected, and the protein concentration was quantified using the BCA reagent (Pierce). The lysates were subjected to SDS‒polyacrylamide gel electrophoresis (PAGE), transferred to a polyvinylidene fluoride (PVDF) membrane, and blocked with 5% bovine serum albumin (BSA) following the manufacturer's instructions. The membranes were incubated with primary antibodies at 4°C overnight. After extensive washing and incubation with the corresponding secondary antibodies, protein bands were developed and captured using an imaging system (UVITEC, Alliance Q9 Advanced Auto). Antibody information is shown in Table S3.

### 2.24 Patient‐Derived Gastric Cancer Organoid Culture and Drug Treatment

Cryopreserved patient‐derived gastric cancer organoids (A2180GPSTL1O106) were purchased from Shanghai CinoBio Biotechnology Co., Ltd. All subsequent recovery, passaging, and maintenance operations were strictly performed in accordance with the manufacturer's official instructions. Briefly, frozen organoid vials were rapidly thawed in a 37°C water bath, followed by centrifugation to remove cryopreservation medium. The recovered organoids were resuspended and embedded in Matrigel, then seeded into 24‐well plates for routine culture. Complete organoid medium was replenished every 2–3 days, and organoids were stably passaged for 2–3 consecutive generations to eliminate cryopreservation‐related cellular stress before drug intervention.

For pharmacological treatment, simvastatin and stiripentol were prepared as stock solutions in dimethyl sulfoxide (DMSO) and further diluted with organoid culture medium to designated working concentrations immediately before use. The final DMSO concentration in all groups was maintained below 0.1% (v/v) to eliminate potential solvent cytotoxicity. Equally sized and morphologically homogeneous organoids were randomly assigned to four groups: vehicle control (0.1% DMSO), 10 µM simvastatin monotherapy, 20 µM stiripentol monotherapy, and combined treatment (10 µM simvastatin + 20 µM stiripentol). The culture medium containing the corresponding drugs was replaced every 3 days. Following continuous treatment for 7 days, bright field imaging was performed, and organoid growth and size were quantitatively analyzed to evaluate the therapeutic effects of single and combined drug administration.

### 2.25 Statistical Analysis

Each experiment was performed at least three times. The sample size for all the other graphs is indicated as “n = x” above the graphs. Statistical analysis was performed using GraphPad Prism 9 software (GraphPad Software, San Diego, CA, USA). The data are expressed as the mean ± standard deviation (SD). Student's *t* test (two‐sided) was used for normally distributed data to compare two groups affected by one variable. The nonparametric Kruskal–Wallis rank‐sum test was used for non‐normally distributed continuous variables. Survival differences were determined using the Kaplan–Meier method and the log‐rank test.

## 3 Results

### 3.1 Characteristics and Prognostic Significance of Protein Lysine Lactylation in GC

To analyze the relationship between Kla expression and GC progression, paired tumor and normal adjacent tissue (NAT) samples were selected from 10 patients with GC according to strict criteria and subjected to lactylome and proteome analyses (Figure 1A and Figure S1A). Global lactylome and proteome analyses were performed using a robust label‐free quantification approach (Figure 1B,C and Figure S1B–E). In total, we identified 5257 Kla sites and 1788 proteins (Figure S1F). Quantitative comparison of the lactyl group between tumors and adjacent tissues revealed that the number of Kla sites and identified proteins significantly increased in GC tumor tissues (Figure 1D and Figure S1F,G). Compared with adjacent tissues, an overall increase in the abundance and type of lactylation modifications was detected in GC tissues (Figure 1E,F, and Figure S1H). Moreover, we employed a pan‐lactylation antibody for immunohistochemical (IHC) staining of GC and adjacent tissues. The results revealed that the intensity of the pan‐lactylation signals was significantly greater in tumor tissues, indicating that Kla modification may be a widely targeted event in GC (Figure 1G). Furthermore, survival analysis indicated that a higher overall Kla intensity within the tumor was significantly associated with a poorer prognosis (Figure 1H). Multivariate Cox regression analysis revealed that high pan‐lactylation (Pan_Kla_group) was not significantly associated with overall survival (HR = 1.89, 95% CI: 0.678–5.267, *P* = 0.2234) (Figure S1I). To gain insight into the amino acid sequence preferences for Kla modifications in human gastric tumor tissues, motif‐X was used to perform Kla motif analysis. IceLogo heatmaps were generated to assess the preference of each residue surrounding the Kla site within a 21‐amino‐acid‐long sequence relative to the human GC proteome (Figure 1I). The heatmaps revealed that alanine, glycine, and lysine residues are present in both upstream and downstream applications and have the highest probability of occurring in lactylated peptides. Meanwhile, cysteine, leucine, and tryptophan residues have the lowest probability of occurring in lactylated peptides. Further analysis also revealed significantly fewer histone Kla sites than nonhistone Kla sites (Figure 1J). Subcellular localization analyses and functional enrichment of lactylated proteins revealed that Kla has a wide range of cellular effects, particularly in the nucleus and cytoplasm (Figure 1K and Figure S1J). These results indicate that GC tissues contain many lactylated proteins.

### 3.2 HADHA K406la Is Elevated in GC Cells

Protein pathway enrichment analyses were subsequently performed to investigate the potential functions of Kla. The results indicated that Kla‐containing proteins were enriched in metabolic, genome stability, and other signature cancer pathways (Figure 2A). Studies have shown that fatty acid metabolism plays a crucial role in regulating tumor adaptation, reprogramming tumor metabolism, and enhancing tumor plasticity [26, 27]. Furthermore, intermediates in fatty acid metabolism also regulate epigenetic modifications in tumors [28, 29, 30]. Therefore, we analyzed lactylation‐modified proteins in fatty acid metabolism pathways and identified five lactylated proteins involved in fatty acid metabolism. Among these lactylation‐modified proteins, HADHA, a key fatty acid metabolism enzyme, plays a significant role in the occurrence and development of various cancers. Notably, HADHA ^K350^ and HADHA ^K406^ were the most frequently lactylated residues (Figure 2B–D, Figure S2A). Therefore, we aimed to validate the function of HADHA ^K350^ and HADHA ^K406^ and further investigate their regulatory and molecular mechanisms. Endogenous and exogenous HADHA were subsequently immunoprecipitated from GC cells and subjected to LC‒MS/MS analysis. The results confirmed pronounced lactylation at the HADHA K406 site (Figure 2E–H), whereas no lactylation was detected at HADHA K350 (Figure S2B). Specific antibodies targeting HADHA K406la and K350la were generated and validated by dot blot assays (Figure S2C–E). Further immunohistochemical staining of GC tissues revealed lactylation at HADHA ^K406^ (Figure 2I). Survival analysis demonstrated that high expression of HADHA K406la was significantly associated with poorer prognosis in patients (Figure 2J). Multivariate Cox regression analysis revealed that high HADHA K406la was a significant independent adverse prognostic factor for overall survival (HR = 2.511, 95% CI: 1.047–6.02, *P* = 0.0391) (Figure S1I). Consistent with this, multiple GC cell lines, along with normal gastric epithelial cells (GES‐1), confirmed the presence of HADHA K406la in GC cells, while negligible modification was observed at the K350 site (Figure 2K,L and Figure S2F). We measured intracellular lactate levels across the same panel of cell lines and found that cellular lactate levels positively correlated with HADHA K406la (Figure S2G). Cell lines with high K406la levels exhibited elevated intracellular lactate levels, whereas GES‑1 cells presented low lactate levels, which is consistent with their weak K406la signal. The small residual K406la detected in GES‑1 cells may reflect metabolic plasticity under culture conditions, such as transient glycolytic activation or local hypoxia, which can result in limited lactate accumulation even in predominantly OXPHOS cells.

Further immunoblotting analysis using HADHA K406la‐ or K350la‐specific antibodies confirmed that lactylation at the K406 site of Myc‐HADHA proteins enabled its immunoprecipitation with an anti‐Myc antibody, whereas HADHA K350 lactylation remained undetectable in GC cells (Figure 2M,N). We speculate that lactylation at HADHA ^K350^ may occur predominantly in nontumor cells within GC tissues. Collectively, these findings demonstrate that lactylation at HADHA K406, rather than at K350, is specifically and highly enriched in GC tumor cells and is clinically relevant to GC progression. Accordingly, we focused on all subsequent functional validation and mechanistic exploration of the K406 lactylation site to systematically elucidate its regulatory role in GC malignant progression.

### 3.3 HADHA K406la Promotes GC Progression by Affecting Energy Metabolism

To better validate the function of HADHA K406la, we generated HADHA‐knockout GC cell lines (Figure S2H) and subsequently rescued these cells with stable overexpression of either HADHA ^WT^ or HADHA ^K406R^ (a lysine‐to‐arginine mutation that abolishes the lactylation site) (Figure S2I). Co‐immunoprecipitation assays confirmed that the HADHA ^K406R^ mutation effectively suppressed HADHA K406la levels (Figure S2J). Posttranslational modifications affect the structure and function of proteins [31, 32]. We used AlphaFold2 to predict the protein structures of HADHA ^WT^ and HADHA ^K406R^ (Figure S2K). Alignment in PyMOL software was then used to compare the protein structures of HADHA ^WT^ and HADHA ^K406R^ (Figure S2L). The alignment results revealed that the conformation of the HADHA ^K406R^ protein changed at the N‐terminus, and HADHA ^K406^ was found to be in the resulting domain of 3‐OH acyl‐CoA, which is located in the HADHA enzyme activity pocket (Figure S2M). To further understand HADHA K406la, we simulated the 3D structure of HADHA K406la (Figure 3A). We subsequently used molecular dynamics simulations to compare the structural dynamics of HADHA^WT^ and HADHA K406la. Docking analysis revealed that the protein helical structure at the lactylated position changed (RMSD = 2.318 Å), and the amino acids in the lactylated region (approximately 350–450 amino acids) underwent significant molecular structural fluctuations (Figure 3B,C). The conformational changes caused by the lactylation of HADHA at K406 likely affect its function. As a nonhistone lactyltransferase, AARS1 was characterized in a cell‐free system supplemented with AARS1, lactyl‐CoA and purified HADHA. Western blotting and mass spectrometry verified K406 lactylation of HADHA upon coincubation with AARS1 and lactyl‐CoA (Figure S2N,O). CRISPR‐Cas9 knockout screening of candidate delactylases revealed that the loss of SIRT1 or SIRT3 significantly downregulated HADHA K406la expression (Figure S2P). Our data indicate that AARS1 and SIRT1/3 are responsible for the lactylation and delactylation of HADHA at K406, respectively.

**FIGURE 3:**
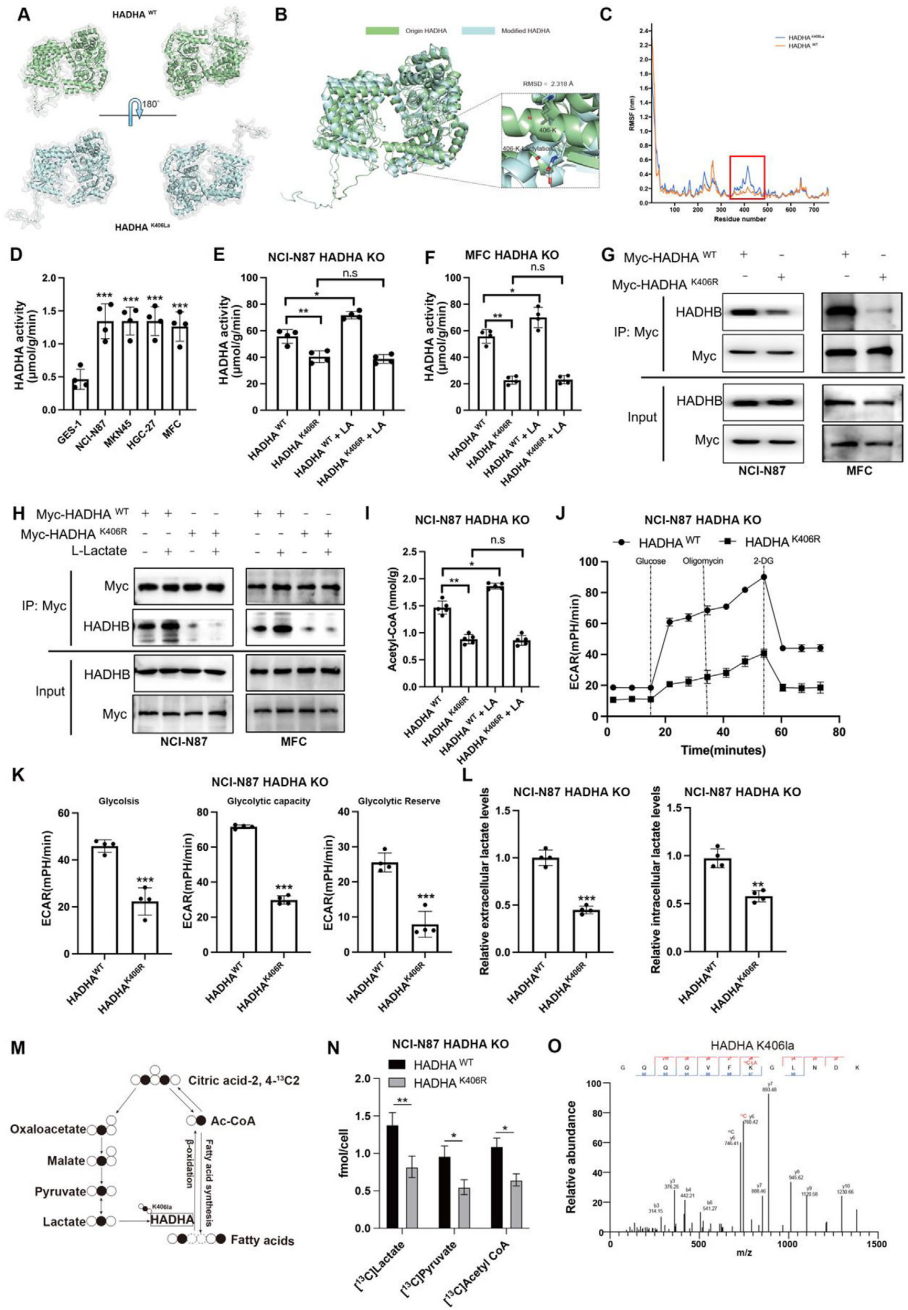
HADHA K406la promotes GC progression. (A) Structures of HADHA^WT^ and HADHA^K406La^. (B) Docking analysis of the structural differences between HADHA^WT^ and HADHA^K406La^. (C) Root mean square fluctuation (RMSF) analysis of molecular fluctuations of amino acids in HADHA^WT^ and HADHA^K406La^ during structural dynamics. (D) Lysates isolated from equivalent numbers of GC and GES cells were used to determine HADHA activity (mean ± SD, n = 4). (E, F) HADHA activity in NCI‐N87 or MFC HADHA KO cells overexpressing HADHA ^WT^ or HADHA ^K406R^ (mean ± SD, n = 4). (G) Co‐immunoprecipitation analysis of the interaction between HADHA^K406R^ and HADHB. (H) Co‐immunoprecipitation analysis of the interaction between HADHA and HADHB in lactate‐treated cells. (I) The effects of HADHA or HADHA^K406R^ on the levels of acetyl‐CoA were analysed in NCI‐87 cells with or without lactate treatment (mean ± SD, n = 4). (J, K) OCRs were used to determine the rate of oxidative phosphorylation in NCI‐N87 HADHA KO cells overexpressing HADHA ^WT^ or HADHA ^K406R^ (mean ± SD, n = 4). (L) Levels of intracellular or extracellular lactate in NCI‐N87 KO cells overexpressing HADHA ^WT^ or HADHA ^K406R^ (mean ± SD, n = 4). (M) Schema of the metabolic pathway illustrating the labelled carbons (black circles) from [2,4‐^13^C] citrate (25 mM; 48 h) to the pyruvate pathway, fatty acid metabolism, and lactate production pathway. (N) Comparison of the levels of [^13^C] lactate, [^13^C] pyruvate and [^13^C] acetyl‐CoA between NCI‐N87 HADHA ^WT^ and NCI‐N87 ^K406R^ (mean ± SD, n = 4). (O) The peak of HADHA K406la is derived from mass spectrometry analysis of [2,4‐13C] citrate‐treated NCI‐N87 cells. *p < 0.05, *p < 0.01, ***p < 0.001.

We subsequently discovered that HADHA activity was significantly higher in cancerous cells than in normal cells (Figure 3D). Moreover, we stably overexpressed wild‐type HADHA (HADHA ^WT^) or the HADHA K406 mutant (HADHA ^K406R^) in HADHA knockout (HADHA KO) NCI‐N87 and MFC cells. Subsequent HADHA activity assays revealed that compared with HADHA ^WT^, HADHA ^K406R^ significantly reduced HADHA activity (Figure 3E,F). Notably, lactic acid treatment of GC cells significantly increased the enzymatic activity of HADHA ^WT^, whereas lactic acid had little effect on the enzymatic activity of HADHA ^K406R^ (Figure 3E,F). Both HADHA and HADHB form mitochondrial trifunctional protein (TFP); therefore, they play important roles in the final step of mitochondrial β‐oxidation of long‐chain fatty acids. We found that the HADHA ^K406R^ mutation significantly inhibited HADHA–HADHB binding (Figure 3G). Furthermore, although lactate enhanced the interaction between wild‐type HADHA and HADHB, it failed to rescue binding in the K406R mutant (Figure 3H). Subsequent analysis of the intracellular acetyl‐CoA levels revealed that HADHA ^K406R^ significantly decreased the intracellular acetyl‐CoA level (Figure 3I). Lactic acid treatment significantly increased the intracellular acetyl‐CoA level in HADHA ^WT^ NCI‐N87 cells but had little effect on the intracellular acetyl‐CoA level in HADHA ^K406R^ cells (Figure 3I). Collectively, these results indicate that HADHA K406la positively regulates fatty acid β‐oxidation by modulating the HADHA protein conformation, enzymatic activity, and complex formation. Furthermore, we analyzed cellular energy metabolism by measuring the oxygen consumption rate (OCR), which reflects mitochondrial oxidative phosphorylation, and the extracellular acidification rate (ECAR), which reflects the glycolytic flux in HADHA KO GC cells rescued with HADHA ^WT^ or HADHA ^K406R^. The results revealed that HADHA K406R expression significantly reduced overall glycolytic activity, including the glycolytic capacity and glycolytic reserve, but did not compensate for the increase in oxidative phosphorylation (Figure 3J,K and Figure S3A,B). We subsequently measured the intracellular and extracellular lactate levels in GC cells expressing HADHA ^WT^ or HADHA ^K406R^. Consistent with our metabolic flux analysis, HADHA ^K406R^ significantly reduced both intracellular and extracellular lactate production (Figure 3L and Figure S3C). The canonical Warburg effect theory suggests that impaired mitochondrial fatty acid β‐oxidation usually triggers compensatory increases in glycolysis and lactate accumulation; however, our results revealed that the loss of HADHA K406la overrides this adaptive metabolic compensation. This unique phenotype is driven by a positive feedback regulatory loop in which HADHA K406la enhances HADHA enzymatic activity, promotes fatty acid β‐oxidation and acetyl‐CoA production, and consequently sustains high glycolytic flux and continuous lactate generation. Disruption of K406 lactylation breaks this forward feedback cycle, leading to a net reduction in glycolysis and lactate production, even under conditions of impaired mitochondrial β‐oxidation.

Consistent with this regulatory model, we further identified a reciprocal feedback loop between HADHA K406la and lactate production in GC cells. We used [2,4‐^13^C]‐labeled citrate‐cultured GC cells to elucidate the direct feedback pathway between HADHA‐mediated lactylation and lactate production in detail (Figure 3M). NCI‐N87 cells were treated with [2,4‐^13^C]‐citrate for 48 h for metabolic flux analysis. Metabolic flux analysis of NCI‐N87 HADHA WT/K406R cells revealed that HADHA ^K406R^ significantly reduced the levels of [^13^C] lactate, [^13^C] pyruvate and [^13^C] acetyl‐CoA (Figure 3N). In addition, the mass spectrometry results also revealed that the lactic acid in the lactylation of HADHA ^K406^ comes from [^13^C] lactate (Figure 3O). Together, these results demonstrate that HADHA K406la promotes lactate production in GC cells and that the newly generated lactate further alters and sustains lactylation at the HADHA K406 site.

Fatty acid metabolism and glycolysis play a role in promoting tumor progression in many tumors; therefore, we also explored the effects of HADHA K406la on tumor progression. The effects of HADHA K406la on the proliferation and migration of GC cells were subsequently assessed using MTT and Transwell assays, respectively. The results revealed that HADHA ^K406R^ significantly inhibited the proliferation, invasion, and migration of GC cells (Figure S3D–I). Moreover, nude mice were injected with HADHA ^WT^, HADHA ^K406R^ NCI‐N87, or MFC GC cells to assess tumor growth. The results revealed that HADHA ^K406R^ expression significantly suppressed the progression of GC (Figure S3J–O). Collectively, these data suggest that HADHA K406la regulates GC progression by modulating intracellular energy metabolism.

To better evaluate the lactylation modification at HADHA ^K406^, we performed site‐directed mutagenesis to validate its functional role. Specifically, we generated the HADHA mutant K406Q (a constitutively lactylated mimetic) and expressed it in HADHA‐knockout GC cells (Figure S4A). Our findings revealed that the K406Q mutation increased HADHA enzymatic activity, increased acetyl‐CoA levels, and promoted GC cell proliferation and invasion (Figure S4B–G). In contrast, the K406R mutation attenuated these effects. These results confirm the site‐specific regulatory role of K406 lactylation in modulating HADHA function and GC progression.

Furthermore, we treated HADHA ^WT^ and HADHA ^K406R^ GC cells with the lactate dehydrogenase (LDH) inhibitor FX11 (5 µM, 48 h) to assess the effect of HADHA K406la on metabolic reprogramming under reduced intracellular lactate levels. Immunoblot analysis confirmed that FX11 treatment markedly decreased HADHA K406la in GC cells (Figure S4H). Functional metabolic detection revealed that FX11 treatment reduced HADHA enzyme activity, decreased the acetyl‐CoA content, and suppressed overall glycolytic phenotypes, including the glycolytic capacity and glycolytic reserve, in HADHA WT cells. In contrast, FX11 had no additional metabolic effects in the lactylation‐deficient K406R cells (Figure S4I–K). In summary, these data demonstrate that HADHA K406la directly enhances HADHA enzymatic activity and drives metabolic reprogramming in GC.

### 3.4 HADHA K406la Drives Transcriptional Activation of HMGCR Through H3K9ac‐Mediated Epigenetic Regulation in GC

To better understand whether HADHA K406la also regulates other metabolic pathways, we first established a GC model using HADHA ^K406R^ cells and collected tumor tissue samples for RNA sequencing. Our findings indicated that, compared with those in the HADHA ^WT^ tissue, several genes involved in lipid metabolism were significantly downregulated in the HADHA ^K406R^ tissue (Figure 4A,B). Furthermore, pathway enrichment analysis of these downregulated genes also revealed a strong correlation between HADHA K406la and lipid metabolism (Figure 4C). We subsequently conducted proteomic analysis of HADHA ^WT^ and HADHA ^K406R^ tissues. Our findings revealed that lipid levels were significantly reduced in HADHA ^K406R^ tissues, with cholesterol showing the most notable decrease (Figure 4D). Hence, to confirm our lipomic findings, we performed Filipin III staining and cholesterol content detection in the tumor tissues. The results clearly indicated that the HADHA ^K406R^ mutation significantly reduced cholesterol levels in tumor tissues (Figure 4E,F). We subsequently constructed mouse models of HADHA ^WT^ and HADHA ^K406R^ GC and fed them a high‐cholesterol diet (HCD) to investigate the role of cholesterol in the progression of GC promoted by HADHA K406la. The results revealed that the HADHA ^K406R^ mutation significantly inhibited the progression of GC, whereas the HADHA ^K406R^ mutation also alleviated the progression of GC caused by a high‐cholesterol diet (Figure 4G–I). These results indicated that HADHA K406la promotes GC progression by increasing cholesterol levels.

**FIGURE 4:**
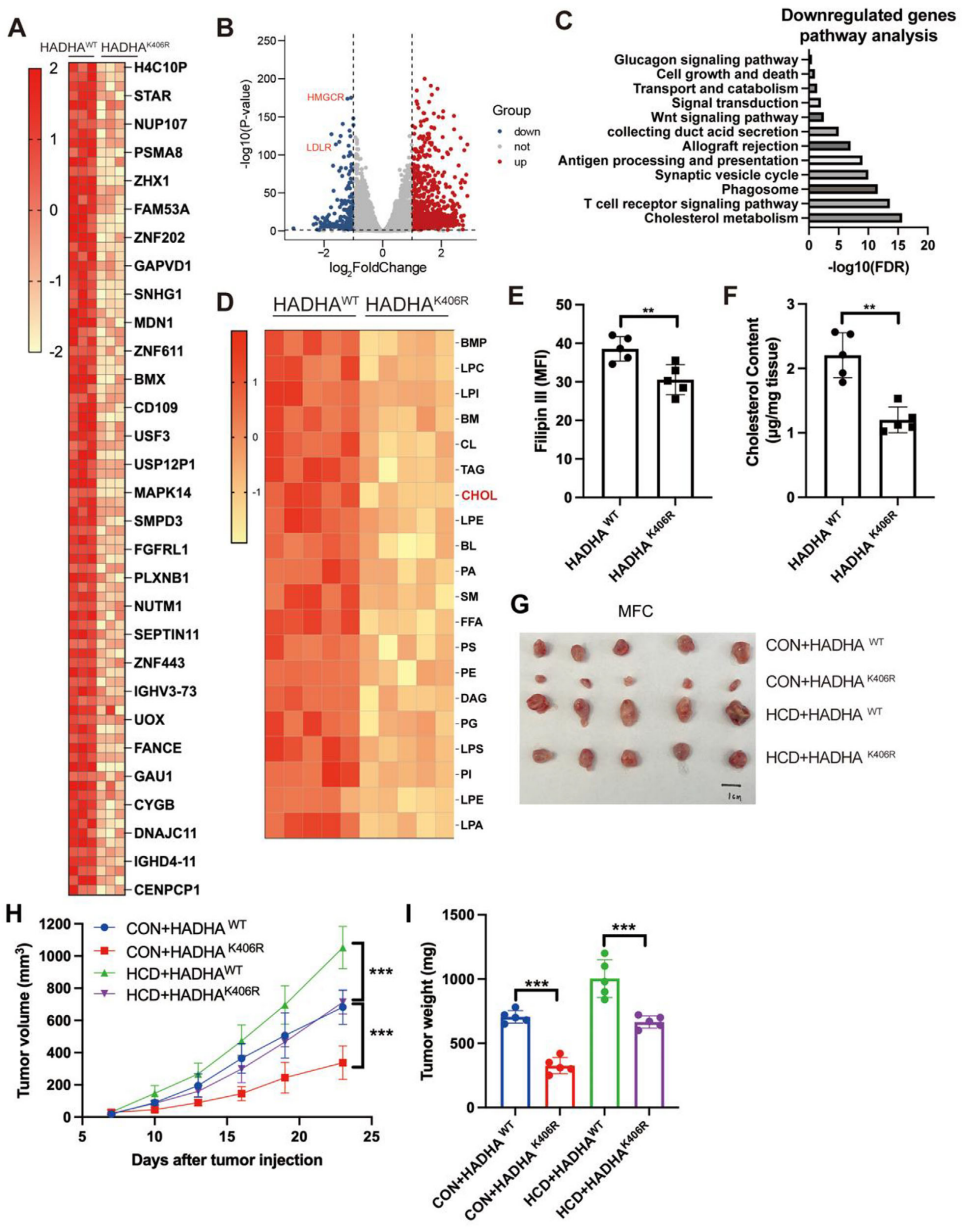
HADHA K406la affects intratumoral CD8^+^ T‐cell function by regulating cholesterol secretion. (A–D) MFC HADHA KO cells overexpressing HADHA ^WT^ or HADHA ^K406R^ were subcutaneously injected into C57BL/6J mice. RNA sequencing (n = 3) and lipidomic analysis (n = 5) were performed on tumor tissues. FC, fold change. FDR, false discovery rate. (A) Heatmap showing the expression levels of downregulated genes. (B) Volcano plot. (C) KEGG enrichment analysis of downregulated genes. (D) Heatmap of changes in lipid species that occurred in HADHA ^K406R^ MFC cells. (E, F) Filipin III staining and cholesterol content in tumor tissues (mean ± SD, n = 3). (G) C57BL/6J mice were fed a high‐cholesterol diet (HCD) for 8 weeks prior to inoculation with MFC HADHA KO cells overexpressing HADHA ^WT^ or HADHA ^K406R^ (scale bar = 1 cm). (H, I) Tumor growth curve and weight in G (mean ± SD, n = 4). *p < 0.05, **p < 0.01 and ***p < 0.001.

To further elucidate how HADHA K406la regulates cholesterol synthesis and given that protein lactylation has been shown to modulate epigenetic modifications [13, 33], we hypothesized that HADHA K406la might influence the epigenetic regulation of genes involved in cholesterol synthesis. Histone acetylation is known to regulate the transcription of multiple genes and thereby affect tumor progression [34, 35, 36]. Our results revealed that HADHA ^K406R^ significantly reduced the intracellular acetyl‐CoA level in GC cells (Figure 5A). Consistent with these findings, western blot analysis demonstrated a marked reduction in global protein acetylation in cells expressing acetylation‐deficient HADHA K406R (Figure 5B). RNA‐seq analysis further revealed no differential expression of histone acetyltransferases (KAT2A, KAT2B, KAT3A, KAT3B, KAT5, KAT6A, KAT6B, KAT7, and KAT8) in HADHA ^K406R^ cells (Figure S5A). Subsequent screening of acetylation levels at specific histone sites revealed that H3K9ac was markedly reduced in HADHA ^K406R^ cells, whereas the levels of H2AK4ac, H2AK5ac, H3K27ac, and H3K36ac remained largely unchanged (Figure 5C). Moreover, HADHA ^K406R^ did not affect other H3K9 modifications, including methylation, lactylation, or butyrylation (Figure S5B). We next performed ChIP‐seq experiments using an H3K9ac antibody in HADHA ^WT^ and HADHA K406R GC cells. The results revealed a significant reduction in H3K9ac abundance in the HADHA ^K406R^ group compared with that in the HADHA WT group (Figure 5D,E). By combining RNA‐seq and H3K9ac ChIP‐seq data from HADHA K406R GC cells, we identified 18 genes whose expression was significantly downregulated. Among these genes, HMGCR, a key rate‐limiting gene closely associated with endogenous cholesterol metabolism biosynthesis, was the most significantly downregulated target (Figure 5F). Subsequent analysis using the Integrative Genomics Viewer (IGV) further confirmed that HADHA ^K406R^ specifically reduces H3K9ac epigenetic modification at the HMGCR promoter region rather than exerting its effect through direct binding to any of these molecules (Figure 5G). Further functional enrichment of the ChIP‐seq differential peaks also revealed that HADHA ^K406R^ significantly inhibited the cholesterol synthesis pathway (Figure 5H). Consistent with the above results, additional ChIP‒qPCR further confirmed that HADHA K406R led to a prominent decrease in H3K9ac enrichment at the HMGCR promoter (Figure 5I). Furthermore, qRT‒PCR and western blotting verified that HADHA ^K406R^ significantly inhibited both the mRNA and protein expression of HMGCR (Figure 5J,K). Supplementation with an acetyl‐CoA precursor (sodium acetate) partially rescued H3K9ac abundance and HMGCR expression in HADHA ^K406R^ cells (Figure 5L). Taken together, these results demonstrate that HADHA K406la indirectly promotes cholesterol synthesis in GC cells by increasing H3K9ac‐mediated epigenetic transcriptional activation of HMGCR.

**FIGURE 5:**
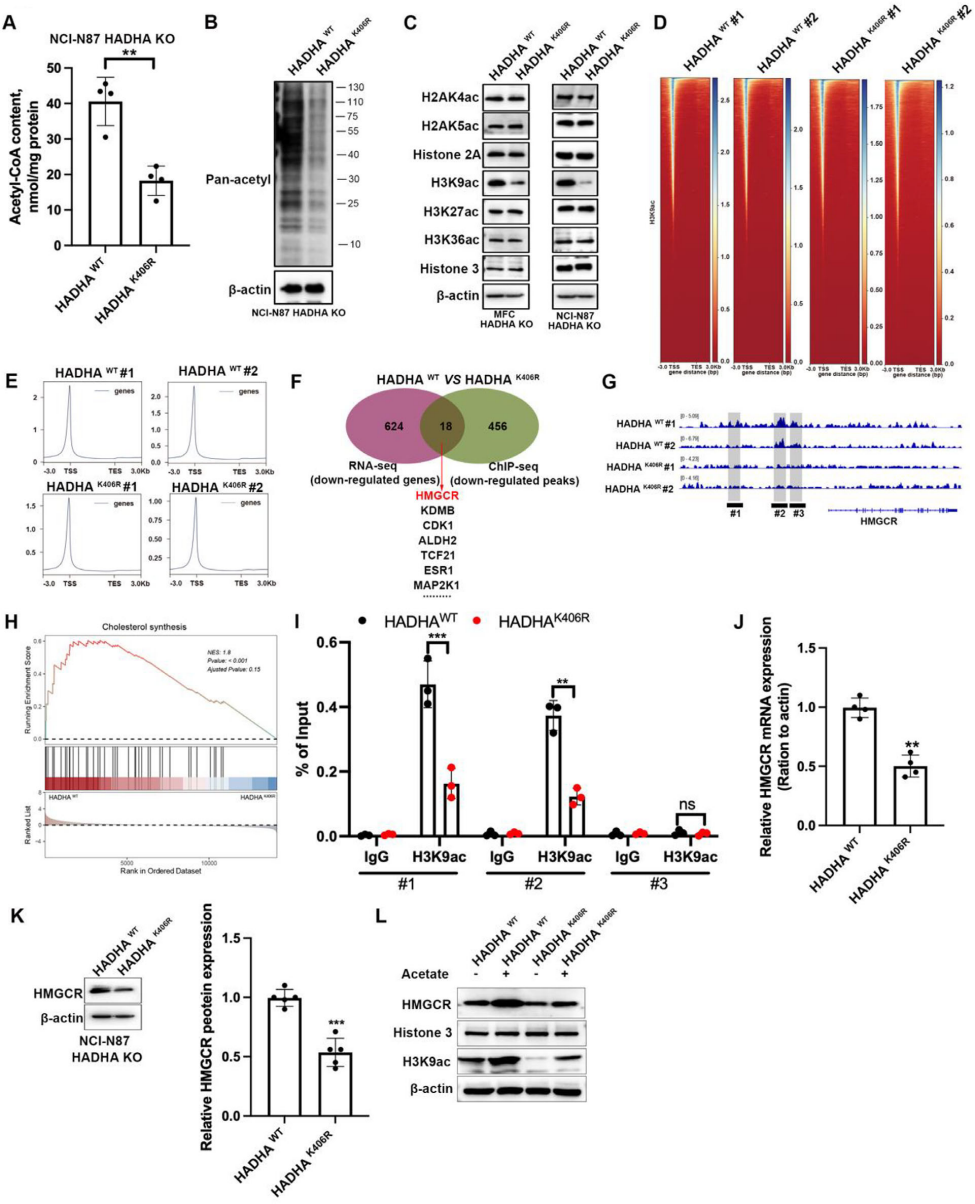
Lactylation of HADHA ^K406^ enhances histone acetylation within the HMGCR promoter region. (A) The content of acetyl‐CoA in HADHA ^WT^ or HADHA ^K406R^ NCI‐N87 cells was analysed (mean ± SD, n = 5). (B) western blot analysis of pan‐acetylation levels in HADHA ^WT^ or HADHA ^K406R^ NCI‐N87 cells. (C) western blot analysis of different levels of histone acetylation (H2AK4ac, H2AK5ac, H3K9ac, H3K27ac, and H3K36ac) in HADHA ^WT^ or HADHA ^K406R^ NCI‐N87 cells and MFC cells. (D, E) Heatmap (D) and profile (E) showing differential peaks in H3K9ac ChIP‐seq analysis of HADHA ^WT^ or HADHA ^K406R^ NCI‐N87 cells. (F) Joint analysis of differentially expressed genes by ChIP‐seq and RNA‐seq. The top genes were selected on the basis of the combined fold‑change and coexpression significance (adjusted p < 0.05). (G) Peak plot showing the binding of HMGCR to H3K9ac. (H) GSEA of the cholesterol synthesis pathway with differential peaks by ChIP‐seq. (I) ChIP‒qPCR analysis of the gene region in which HADHA binds to HMGCR (mean ± SD, n = 3). (J, K) qRT‒PCR and immunoblotting experiments were used to analyse the expression level of HMGCR in HADHA ^WT^ or HADHA ^K406R^ NCI‐N87 cells (mean ± SD, n = 4). (L) Effects of sodium acetate supplementation on the acetyl‐CoA level and H3K9ac expression in HADHA ^WT^ and HADHA ^K406R^ GC cells. **p < 0.01, ***p < 0.001.

To further elucidate the regulatory axis linking HADHA K406la to H3K9ac‐dependent *HMGCR* transcription, we performed CRISPR screening to knockout individual histone acetyltransferases (KATs). Compared with the other KATs, KAT2A knockout most profoundly suppressed *HMGCR* expression (Figure S5C). Consistent with these findings, KAT2A has been established as the primary histone acetyltransferase responsible for H3K9 acetylation, which drives gastric cancer progression via H3K9ac [37, 38]. We further confirmed that KAT2A knockdown markedly reduced H3K9ac levels in HADHA ^WT^ cells but had a minimal effect on H3K9ac in HADHA ^K406R^ cells (Figure S5D). Accordingly, KAT2A knockout significantly decreased *HMGCR* expression and cholesterol synthesis in HADHA WT cells but had no such effect in HADHA K406R cells (Figure S5D, E). ChIP‐seq further confirmed that KAT2A directly binds the *HMGCR* promoter and that HADHA ^K406R^ substantially attenuates KAT2A recruitment to this region (Figure S5F–H). Collectively, these data demonstrate that HADHA K406la modulates global H3K9ac by governing acetyl‐CoA availability in a KAT2A‐dependent manner, thereby increasing H3K9ac enrichment at the *HMGCR* promoter, promoting *HMGCR* transcription, and driving cholesterol synthesis.

Previous studies have shown that HMGCR expression is regulated through multiple pathways. For example, methylated RIG‐I interacts with AMPKα to promote cholesterol synthesis by inhibiting HMGCR phosphorylation and enhancing its enzymatic activity [39]. Additionally, CRTC2 and SERBP‐2 transcriptionally regulate HMGCR expression [40, 41]. We therefore investigated whether HADHA K406la also regulates HMGCR through these alternative mechanisms. Our results indicated that HADHA K406R did not affect the expression or interaction of RIG‐I and AMPKα (Figure S5F,G). Moreover, HADHA K406R did not alter the expression of CRTC2 or SERBP‐2 (Figure S5H,I). These data suggest that HADHA K406la regulates HMGCR expression independently of the AMPKα and SERBP‐2 pathways.

### 3.5 Single‐Cell Analysis of the Immune Microenvironment in HADHA^WT^ and HADHA^K406R^ GC Tumors

Given that tumor‐derived cholesterol is capable of remodeling intratumoral immune cell function [42, 43], we sought to determine whether HADHA K406la modulates the immune microenvironment of GC. To this end, we subcutaneously implanted MFC‐HADHA‐knockout cells stably expressing either HADHA WT or HADHA K406R into C57BL/6J mice and performed single‐cell RNA sequencing (scRNA‐seq) to characterize the immune landscape within GC tumors (Figure S6A). A total of 32 863 cells were isolated from tumor tissues via fluorescence‐activated cell sorting (FACS). After stringent quality filtering (unique RNA features > 10 000 and a mitochondrial gene ratio < 20%), 25,639 high‐quality cells were retained for subsequent analysis. Unsupervised clustering revealed nine major immune and stromal cell populations, including T cells (*Ptprc* and *Cd3d*) [44], B cells (*Cd79a, Cd79b* and *Igkc*) [45], plasmacytoid dendritic cells (pDCs; *Runx2, Bcl11a, Tcf4* and *Siglech*) [46, 47, 48, 49], conventional dendritic cells (cDCs; *Batf3, Cbfa2t3, Ccl5* and *Ccl22*) [50, 51], antigen‐presenting cells (APCs; *H2‐Aa, H2‐Ab1, H2‐Eb1* and *Cd74*) [52, 53, 54], neutrophils (*Ly6g, Ltf* and *Lcn2*) [55, 56, 57, 58], polymorphonuclear myeloid‐derived suppressor cells (PMN‐MDSCs; *Hdc, Ccl3, Ccr1* and *Cxcr2*) [59, 60, 61, 62], Chil3‐monocytes (*Chil3, Lyz2* and *Ly6c2*) [63], and macrophages (*C1qa, C1qb, Fn1, Lrp1, Mrc1, Arg1* and *Il10*) [64] (Figure S6B–D). Our comparative analysis revealed that compared with the controls, the HADHA ^K406R^ GC group had more inflammation‐associated T cells and fewer macrophages (Figure S6E, F and Figure S7A,B), suggesting that T‐cell function may play a role in the ability of the HADHA K406la modification to promote GC progression.

To further elucidate T‐cell heterogeneity and transcriptional alterations, we reclustered T cells and identified nine distinct subpopulations: mitotic T cells (*Mki67 and Stmn1*) [65], naive CD4^+^ T cells (*Il7r* and *Tcf7*) [66], Th17 cells (*Rora, Tnfrsf4* and *Hif1a*) [67], Treg cells (*Ctla4, Foxp3* and *Il2ra*) [68], naive CD8 T cells (*Ccr7* and *Sell*) [65], exhausted CD8^+^ T cells (Tex; *Pdcd1, Lag3* and *Havcr2*) [69], Gzmk effector CD8^+^ T cells (*Gzmk, Ccl3, Ccr1* and *Cxcr2*) [66], mitotic CD8^+^ T cells (*Mki67* and *Stmn1*) [65], and NK cells (*Gzma, Nkg7, Klra7, Ccl5* and *Xcl1*) [65] (Figure 6A,B). Given the regulatory effect of HADHA K406la on HMGCR‐mediated cholesterol synthesis, we next focused on cholesterol metabolic reprogramming in intratumoral CD8^+^ T cells. Module scoring analysis revealed that CD8^+^ T cells from HADHA ^K406R^ tumors displayed markedly decreased signatures for cholesterol uptake (*Ldlr, Vldlr, Lrp1, Scarb1, Cd36, Npc1* and *Npc2*), cholesterol synthesis (*Hmgcr, Hmgcs1, Mvk, Mvd, Fdps, Sqle, Lss, Dhcr7, Dhcr24, Srebf2, Insig1* and *Insig2*), and cholesterol‐dependent cellular function (*Ddit3, Atf4, Atf3, Xbp1, Hspa5, Bcl2l11, Bbc3, Tox, Nr4a1, Nr4a2* and *Nr4a3*). In contrast, these cells exhibited elevated cholesterol efflux activity (*Abca1, Abcg1, Apoe, Apoa1, Nr1h3* and *Nr1h2*) (Figure S7C–G). Collectively, these findings indicate that disruption of HADHA K406la globally suppresses cholesterol metabolism in tumor‐infiltrating CD8^+^ T cells.

**FIGURE 6:**
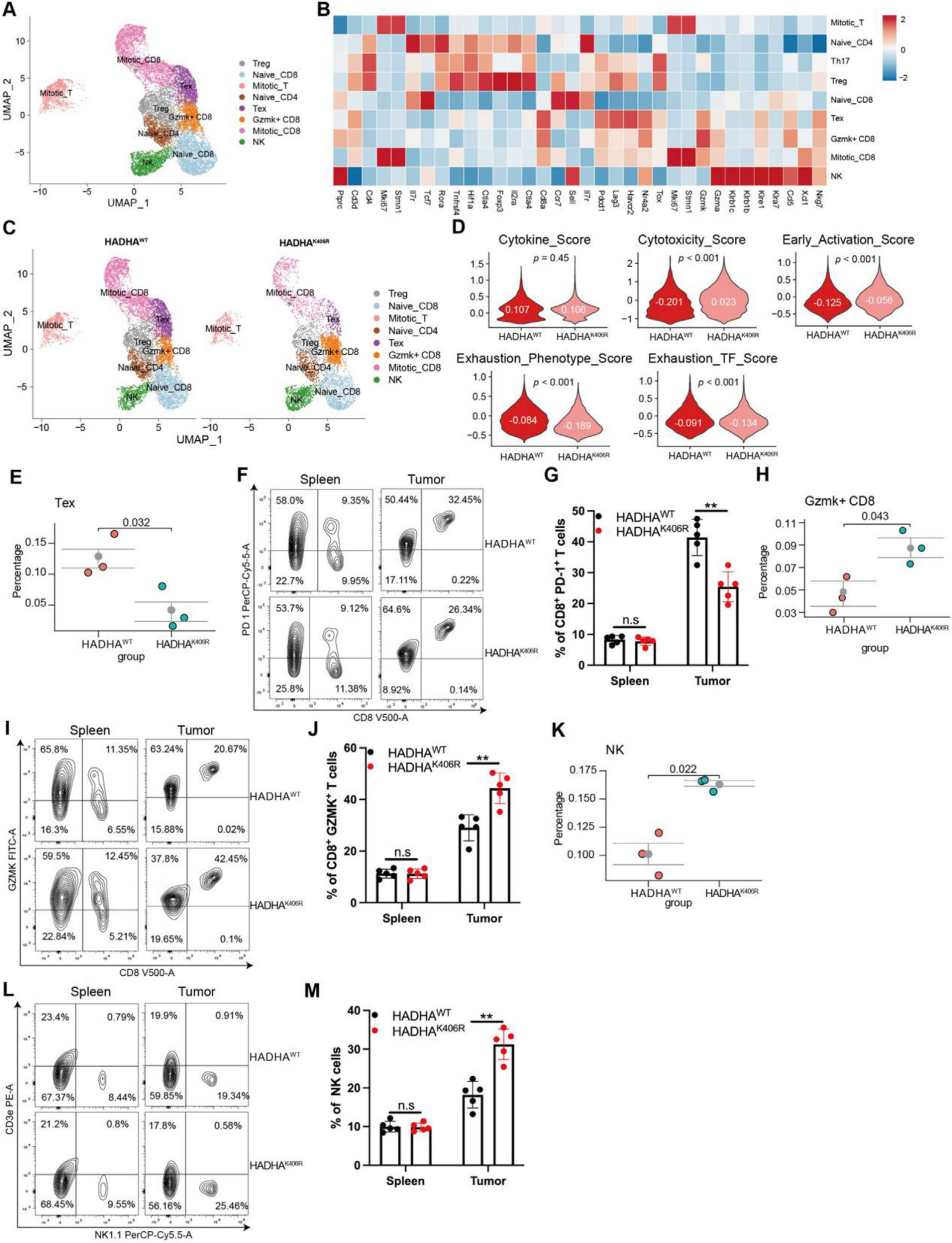
HADHA K406R remodels the immunosuppressive tumor microenvironment in gastric cancer. (A) UMAP plot visualizing 9 distinct T‐cell subsets identified from the scRNA‐seq dataset. (B) Heatmap showing the average expression of canonical marker genes across the 9 T‐cell populations. (C) UMAP plot of the 9 T‐cell subsets, batch‐corrected using the Harmony algorithm, comparing tumors from the HADHA ^K406R^ and HADHA ^WT^ groups. (D) AddModuleScore analysis comparing gene signature scores for cytokine production, cytotoxicity, exhaustion‐associated transcription factors, early T‐cell activation, and T‐cell exhaustion in CD8^+^ T cells from HADHA ^WT^ and HADHA ^K406R^ tumors. (E) Frequencies of exhausted CD8^+^ T cells in HADHA ^K406R^ tumors compared with those in HADHA ^WT^ tumors, as quantified from the scRNA‐seq data. (F, G) Flow cytometric analysis showing the frequency of PD‐1^+^ CD8^+^ T cells among total CD8^+^ T cells in the spleen and among tumor‐infiltrating CD8^+^ T cells from HADHA ^K406R^ and HADHA ^WT^ tumor‐bearing mice (mean ± SD, n = 5). (H) Frequencies of GZMK^+^ CD8^+^ T cells in HADHA ^K406R^ tumors versus those in HADHA ^WT^ tumors, as quantified from the scRNA‐seq data. (I, J) Flow cytometric analysis showing the frequency of GZMK^+^ CD8^+^ T cells among total CD8^+^ T cells in the spleen and among tumor‐infiltrating CD8^+^ T cells from HADHA ^K406R^ and HADHA ^WT^ tumor‐bearing mice (mean ± SD, n = 5). (K) Frequencies of NK cells in HADHA ^K406R^ tumors versus those in HADHA ^WT^ tumors, as quantified from the scRNA‐seq data. (L, M) Flow cytometric analysis showing the frequency of NK cells among total immune cells in the spleen and among tumor‐infiltrating immune cells from HADHA K406R and HADHA WT tumor‐bearing mice (mean ± SD, n = 3). **p < 0.01.

We further systematically evaluated functional gene sets associated with CD8^+^ T‐cell effector properties, including cytokines (*Ifng*, *Tnf*, *Il2*, *Il4*, *Il10*, *Il17a*, *Il21*, *Cst2*, *Ccl3*, *Ccl4*, *Ccl5* and *Xcl1*), cytotoxic molecules (*Gzma*, *Gzmb*, *Gzmk*, *Prf1* and *Nkg7*), early activation markers (*Cd69*, *Il2ra*, *Cd38*, *Cd40lg*, *H2‐Aa* and *H2‐Ab1*), exhaustion‐related surface receptors (*Pdcd1*, *Ctla4*, *Lag3*, *Havcr2*, *Tigit*, *Btla*, *Vsir*, *Entpd1*, *Cd38* and *Lgals3*), and exhaustion‐associated transcription factors (*Tox*, *Nr4a1*, *Nr4a2*, *Nr4a3*, *Eomes*, *Prdm1*, *Tbx21* and *Foxp1*). Notably, intratumoral CD8^+^ T cells from HADHA ^K406R^ tumors exhibited markedly elevated cytotoxicity and early activation scores, alongside reduced signatures of T cell exhaustion (Figure 6D and Figure S7H–L). In line with these transcriptional alterations, the proportion of exhausted CD8^+^ T (Tex) cells was markedly reduced, whereas the frequencies of Gzmk effector CD8^+^ T cells and NK cells were increased in the HADHA ^K406R^ group (Figure 6E–M). Flow cytometry further validated these scRNA‐seq findings at the protein level (Figure 6E–M). Taken together, these single‐cell sequencing and flow cytometric results demonstrate that HADHA K406la drives cholesterol metabolic dysregulation in intratumoral CD8^+^ T cells, ultimately inducing CD8^+^ T‐cell exhaustion and establishing an immunosuppressive tumor microenvironment.

### 3.6 HADHA K406la  Regulates the Functions of CD8^+^ T Cells and Myeloid Cells in GC

Studies have shown that cholesterol induces CD8^+^ T‐cell exhaustion in the tumor microenvironment, thereby inhibiting host antitumor immunity [42]. Therefore, we conducted a thorough analysis of the quantity and condition of CD8^+^ T cells within the GC tissues of mice harbouring either MFC‐HADHA ^WT^ or MFC‐HADHA ^K406R^. The results revealed that HADHA ^K406R^ expression significantly increased the number of infiltrating CD8^+^ T cells (Figure 7A). MCF HADHA KO cells stably overexpressing HADHA ^WT^ or HADHA ^K406R^ were subsequently cocultured with mouse CD8^+^ T cells, after which the mouse CD8^+^ T cells were collected for RNA‐seq. The results revealed that HADHA ^K406R^ significantly upregulated the expression levels of the proliferation‐related gene Ki67 [70] and the recruitment‐related genes Ccr5 and Cxcr3 [71] in CD8^+^ T cells, whereas the expression of the intracellular apoptosis‐related genes Fas, Tnf, Gas2 and Tnfrs25 [70] was significantly reduced (Figure 7B). Furthermore, CD8^+^ T cells cocultured with HADHA ^WT^ or HADHA ^K406R^ MFC cells were collected, and flow cytometry and qRT‒PCR analyses demonstrated that coculture with HADHA ^K406R^ MFC cells led to a significant reduction in apoptosis, along with increased proliferation and chemokine expression in CD8^+^ T cells (Figure 7C–E). Additionally, we assessed the state of CD8^+^ T cells in tumors, spleens, and lymph nodes from mice bearing HADHA ^WT^ or HADHA ^K406R^ MFC tumors. Flow cytometry revealed that HADHA ^K406R^ markedly increased IFN‐γ and TNF expression in CD8^+^ T cells while substantially reducing PD‐1 expression (Figure 7F,G). We further analyzed the correlation between HADHA K406la and CD8^+^ T‐cell function using clinical specimens from gastric cancer patients. We found that CD8^+^ T cells in tissues with high HADHA K406la expression displayed elevated PD‐1 levels and markedly decreased IFN‐γ and TNF expression (Figure S8A–C). These results suggest that HADHA ^K406R^ increases CD8^+^ T‐cell immune responses in GC by affecting CD8^+^ T‐cell activation, recruitment, proliferation, and apoptosis.

**FIGURE 7:**
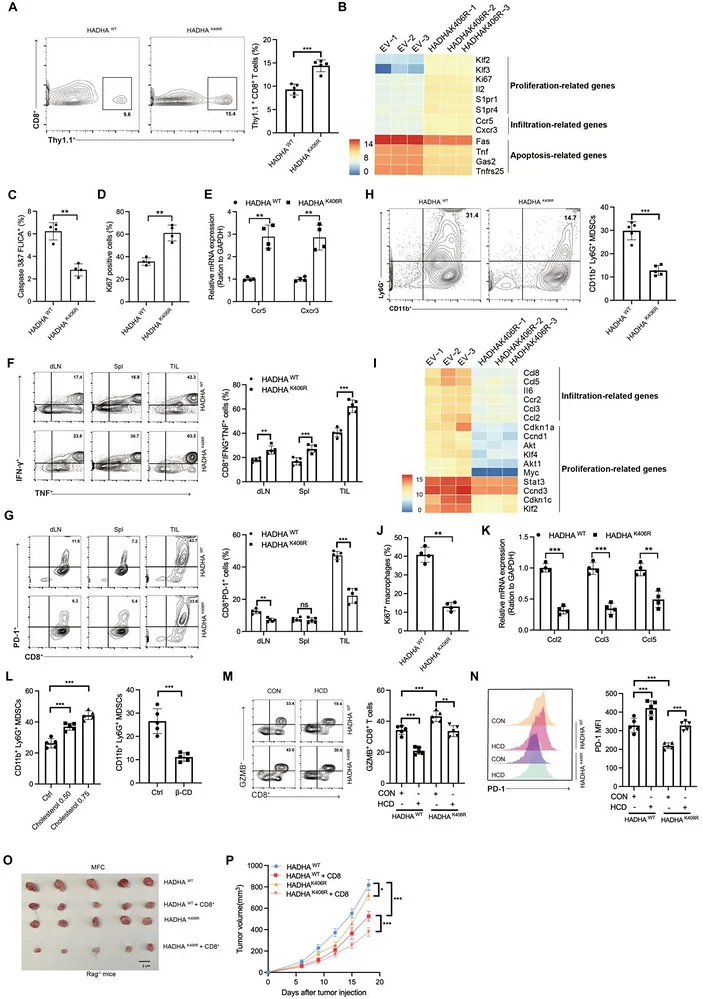
HADHA ^K406^ regulates the functions of CD8^+^ T cells and myeloid cells in GC. (A) The percentage of tumor‐infiltrating CD8^+^ T cells among total tumor‐infiltrating leukocytes in HADHA ^WT^ or HADHA ^K406R^ tumors was analyzed by flow cytometry (mean ± SD, n = 5). (B) MFC HADHA KO cells stably overexpressing HADHA ^WT^ or HADHA ^K406R^ were cocultured with mouse CD8^+^ T cells, and the mouse CD8^+^ T cells were collected for RNA‐seq. Heatmap analysis of the expression levels of genes related to infiltration, proliferation, and apoptosis in CD8^+^ T cells. (C–E) Flow cytometry analysis of the expression levels of caspases 3 and 7 (C), Ki67 (D), Ccr5, and Cxcr3 (E) in the CD8^+^ T cells in B (mean ± SD, n = 4). (F, G) The levels of GZMB, IFNG (F) and PD‐1 (G) in the spleen, draining lymph nodes, and tumor‐infiltrating CD8^+^ T cells in HADHA ^WT^ and HADHA ^K406R^ tumor model mice were analyzed by flow cytometry (mean ± SD, n = 5). (H) The percentage of CD11b^+^Ly6G^+^ cells among total tumor‐infiltrating leukocytes in HADHA ^WT^ or HADHA ^K406R^ tumors was analyzed by flow cytometry (mean ± SD, n = 5). (I) MFC HADHA KO cells stably overexpressing HADHA ^WT^ or HADHA ^K406R^ were cocultured with small MDSCs, and MDSCs from the mice were collected for RNA‐seq. Heatmap analysis of the expression levels of genes related to infiltration and proliferation in MDSCs. (J, K) Flow cytometry analysis of the expression levels of Ki67 (J), Ccl2, Ccl3, and Ccl5 (K) in MDSCs in I (mean ± SD, n = 4). (L) Bone marrow cells isolated from the femurs of C57BL/6J mice were cultured in RPMI 1640 medium supplemented with 10 ng/mL GM‐CSF. On day 3, the cells were treated with 0.75 mg/mL cholesterol for 3 days or 5 mM β‐CD, a cholesterol‐depleting agent, for 24 h. The percentage of MDSCs was determined by flow cytometry (mean ± SD, n = 5). (M, N) The percentages of tumor‐infiltrating GZMB^+^ CD8^+^ T cells (M) and PD‐1^+^ T cells (N) among the total tumor‐infiltrating leukocytes shown in Figure 4I were analysed by flow cytometry (mean ± SD, n = 5). (O, P) HADHA ^WT^ or HADHA ^K406R^ GC cells were injected subcutaneously into Rag^−/−^ mice with or without CD8^+^ T cells from C57BL mice. The tumor growth curves are shown (mean ± SD, n = 5, scale bar = 1 cm). *p < 0.05, **p < 0.01 and ***p < 0.001.

Furthermore, XBP1 intrinsic to cancer cells drives immunosuppressive reprogramming of intratumoral myeloid cells by promoting cholesterol production [43]. Furthermore, analysis of subcutaneous tumor tissues revealed a significant reduction in CD11b^+^ Ly6G^+^ myeloid cells in mice bearing MFC‐HADHA ^K406R^ cells compared with those bearing MFC‐HADHA ^WT^ cells (Figure 7H). MFC HADHA KO cells stably overexpressing HADHA ^WT^ or HADHA ^K406R^ were co‐cultured with MDSCs (myeloid‐derived suppressor cells, MDSCs), and the MDSCs were collected for RNA‐seq. HADHA ^K406R^ significantly reduced the expression levels of the proliferation‐related genes Cdkn1a, Ccnd1, Akt1, Myc, Stat3, Ccnd3, Cdkn1c and Klf2 [72], as well as the recruitment‐related genes Ccr2 and Ccl2/3 [73], in MDSCs (Figure 7I). Similarly, MDSCs cocultured with HADHA ^WT^ or HADHA ^K406R^ MFC cells were harvested, and flow cytometry and qRT‒PCR confirmed that coculture with HADHA ^K406R^ MFC cells resulted in significantly reduced proliferation and chemokine expression in MDSCs (Figure 7J–K). These results indicate that HADHA ^K406R^ decreases the number of MDSCs in GC by affecting MDSC recruitment and proliferation.

To further investigate the effects of cholesterol on CD8^+^ T cells and myeloid cells, we extracted mouse bone marrow cells and CD8^+^ T cells for culture in vitro and treated them with cholesterol and/or β‐cyclodextrin (β‐CD). The results revealed that the cholesterol content was positively correlated with the level of PD‐1 in CD8^+^ T cells and the number of CD11b^+^ Ly6G^+^ myeloid cells (Figure 7L and Figure S8D). Moreover, cholesterol depletion using β‐CD significantly reduced the numbers of exhausted CD8^+^ T cells and CD11b^+^ Ly6G^+^ myeloid cells (Figure 7L and Figure S8E). More importantly, HADHA ^K406R^ upregulated GZMB levels in CD8^+^ T cells, whereas a high‐cholesterol diet partially suppressed this effect of HADHA ^K406R^ mutation (Figure S7M). In addition, a high‐cholesterol diet partially eliminated the inhibitory effect of HADHA ^K406R^ on the PD‐1 level in CD8^+^ T cells (Figure 7N). To further verify the above results, simvastatin was subsequently used to inhibit cholesterol metabolism in HADHA ^WT^ tumors, and the HADHA ^K406R^ mutation upregulated the GZMB level in tumor‐infiltrating CD8^+^ T cells and inhibited the expression of PD‐1 (Figure S8F–J). Furthermore, we constructed subcutaneous tumor models in immunodeficient Rag^−/−^ mice and injected them with CD8^+^ T cells. Injection of CD8^+^ T cells into HADHA WT tumor mice did not significantly inhibit tumor progression, whereas injection of CD8^+^ T cells significantly inhibited HADHA ^K406R^ tumor progression (Figure 7O,P). These results suggest that the lactylation of HADHA ^K406^ promotes cholesterol secretion, thereby inhibiting CD8^+^ T‐cell antitumor immunity and the recruitment of bone marrow‐derived immunosuppressive cells.

### 3.7 HADHA K406la Activates MDSCs via HMGCR‐Cholesterol‐STAT3

Studies have shown that cholesterol promotes MDSC expansion and activation by upregulating STAT3 phosphorylation, iNOS expression, and intracellular reactive oxygen species (ROS) levels [43]. The results of the coculture of MDSCs with MFC HADHA KO cells stably overexpressing HADHA ^WT^ or HADHA ^K406R^ showed that HADHA ^K406R^ significantly inhibited the phosphorylation level of STAT3 in MDSCs (Figure S9A). In addition, HADHA ^K406R^ reduced iNOS mRNA and intracellular reactive oxygen species (ROS) levels in MDSCs (Figure S9B,C). Interestingly, coculture experiments revealed that compared with MDSCs treated with HADHA ^WT^, MDSCs treated with HADHA ^K406R^ alleviated the inhibition of CD8^+^ T‐cell activation in vitro (Figure S9D). MDSCs in which STAT3 or iNOS was knocked down in vitro were cocultured with MFC HADHA KO cells stably overexpressing HADHA ^WT^ or HADHA ^K406R^, respectively, and the results revealed that MDSCs with STAT3 or iNOS knockdown significantly inhibited the number of M2 macrophages in HADHA ^WT^ or HADHA ^K406R^. Notably, coculture of MDSCs with HADHA ^K406R^ suppressed the M2 effect more significantly (Figure S9E,F). In addition, we further explored whether HADHA K406la regulates M2 macrophage differentiation. Consistent with these in vitro findings, we further quantified the intratumoral M2 macrophage proportion in HADHA‐WT and HADHA‐K406R tumor tissues via flow cytometry. The results confirmed that HADHA ^K406R^ markedly inhibited M2 macrophage polarization in vivo (Figure S9G). These results indicate that HADHA K406la activates MDSC activity through HMGCR–cholesterol–STAT3 signaling and increases immunosuppression in GC.

### 3.8 HADHA K406la Inhibits CD8^+^ T‐Cell Antitumor Immunity via the HMGCR‐Cholesterol Core Regulatory Axis

Reprogramming cellular cholesterol metabolism impairs the antitumor immune function of intratumoral CD8^+^ T cells, and HADHA K406la epigenetically drives the transcriptional upregulation of HMGCR, the rate‐limiting enzyme in cholesterol synthesis. We therefore hypothesized that HADHA K406la suppresses CD8^+^ T‐cell antitumor immunity primarily through the downstream HMGCR–cholesterol regulatory axis. We first constructed GC cell lines with stable HMGCR knockdown or overexpression and validated the efficient regulation of intracellular cholesterol levels by HMGCR (Figure S10A,B). Primary mouse CD8^+^ T cells were isolated and cultured in vitro for functional and phenotypic validation. CD8^+^ T cells were treated with conditioned supernatant collected from GC cells transfected with empty vector (EV) or shHMGCR, and flow cytometry was used to assess T‐cell exhaustion and effector function. Notably, the expression of the exhaustion marker PD‐1 was markedly decreased in the shHMGCR group, whereas the expression of the effector molecules GZMB and IFN‐γ was significantly upregulated in CD8^+^ T cells (Figure S10C,D). In contrast, CD8^+^ T cells exposed to supernatant from HMGCR‐overexpressing GC cells exhibited the opposite exhausted and functional phenotypes (Figure S10E,F). To exclude the confounding effects of other secreted factors and clarify whether cholesterol alone is sufficient to drive CD8^+^ T‐cell exhaustion, we cultured CD8^+^ T cells with purified cholesterol under defined conditions. Flow cytometry analysis of PD‐1 expression confirmed the direct regulatory role of cholesterol in inducing CD8^+^ T‐cell exhaustion (Figure S10G). In parallel, exogenous cholesterol supplementation in CD8^+^ T cells cultured with shHMGCR‐derived supernatant markedly restored PD‐1 expression, directly demonstrating that cholesterol accumulation is a critical driver of CD8^+^ T‐cell exhaustion (Figure 8A). Consistently, β‐CD‐mediated cholesterol depletion in CD8^+^ T cells cultured with HMGCR‐overexpressing supernatant effectively reversed the upregulation of PD‐1 expression induced by HMGCR‐driven cholesterol elevation (Figure 8B). We further established a subcutaneous tumor model by inoculating C57BL/6J mice with EV or shHMGCR MFC GC cells. In vivo results revealed that HMGCR knockdown markedly restrained tumor growth, decreased PD‐1 expression in intratumoral CD8^+^ T cells, and increased the infiltration of functional GZMB^+^ and IFN‐γ^+^ CD8^+^ T cells in patients with GC (Figure 8C,D and Figure S10H). In contrast, HMGCR overexpression significantly promoted GC malignant progression, exacerbated CD8^+^ T‐cell exhaustion with elevated PD‐1 levels, and suppressed the expression of key effector cytokines in intratumoral CD8^+^ T cells (Figure 8E,F and Figure S10I). Collectively, these in vitro and in vivo findings confirm that the HMGCR–cholesterol signaling cascade serves as the core downstream mechanism through which HADHA K406la mediates CD8^+^ T‐cell immune suppression.

**FIGURE 8:**
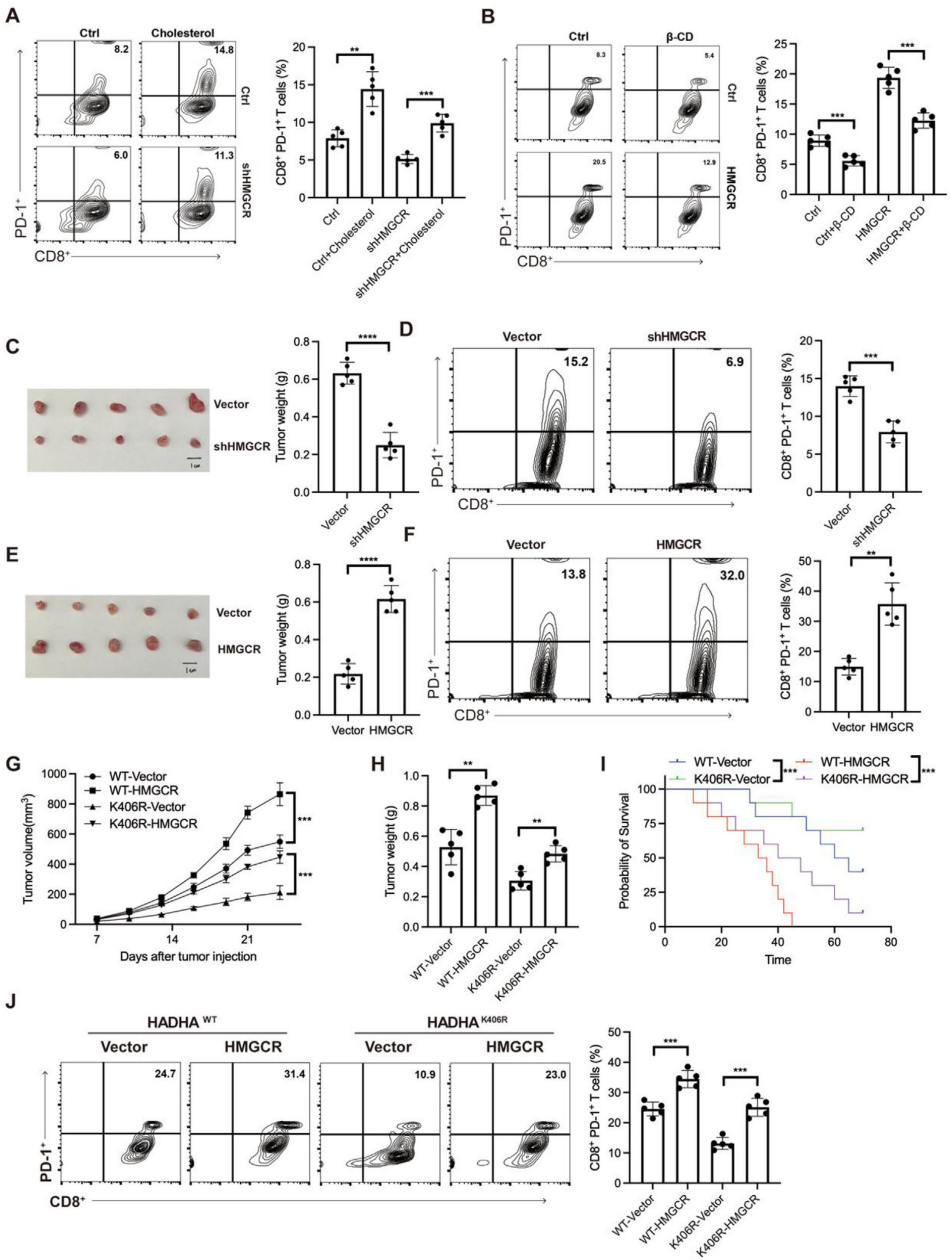
HADHA K406la regulates CD8^+^ T‐cell antitumor immunity via HMGCR‐ cholesterol. (A) CD8^+^ T cells were isolated from C57BL/6J mice and stimulated in vitro with anti‐CD3/CD28 antibodies in the presence of IL‐2. The tumor supernatant (1 mg of tumor in 1 mL of T‐cell culture medium, filtered) was obtained from HMGCR or Ctrl (EV) MFC tumors. CD8^+^ T cells were cultured with 100 mL of EV or shHMGCR tumor supernatant with or without cholesterol as indicated, and the expression of PD‐1 on the cultured CD8^+^ T cells was determined (mean ± SD, n = 5). (B) CD8^+^ T cells stimulated with anti‐CD3/CD28 antibodies in the presence of IL‐2 were cultured with 100 mL of Ctrl (EV) or HMGCR tumor supernatant with or without β‐cyclodextrin (β‐CD, 0.5 mM) as indicated, and the expression of PD‐1 on the cultured T cells was determined (mean ± SD, n = 5). (C–F) 5 × 10^5^ shHMGCR (HMGCR) or Ctrl (vector) MFC cells were subcutaneously injected into C57BL mice (n = 5). Images of the tumors and tumor weights are shown (C, E). The expression of PD‐1 on tumor‐infiltrating CD8^+^ T cells was determined (D, F) (mean ± SD, scale bar = 1 cm). (G–I) 5 × 10^5^ HADHA ^WT^ and HADHA ^K406R^ GC cells treated with or without HMGCR‐overexpressing viruses were subcutaneously injected into C57BL mice (n = 6). Tumor growth curve (G), tumor weight (H) and mouse survival curve (I) (mean ± SD). (J) HADHA ^WT^ and HADHA ^K406R^ GC cells treated with or without HMGCR‐overexpressing viruses were injected subcutaneously into mice. The expression of PD‐1 and Filipin III in tumor‐infiltrating CD8^+^ T cells was detected (mean ± SD, n = 5). **p < 0.01, ***p < 0.001 and ****p < 0.0001.

We further performed in vivo rescue experiments by subcutaneously injecting mice with HADHA ^WT^ or HADHA ^K406R^ GC cells with stable HMGCR overexpression. Tumor growth kinetics and mouse survival were continuously monitored, and the results revealed that HMGCR overexpression largely abrogated the tumor‐suppressive and immune‐repairing phenotypes induced by HADHA ^K406R^ (Figure 8G–I). More importantly, HMGCR overexpression restored intracellular cholesterol accumulation in HADHA K406R tumors, consequently increasing PD‐1 expression and reducing GZMB and IFN‐γ levels in intratumoral CD8^+^ T cells (Figure 8J and Figure S10J). Taken together, these findings demonstrate that HADHA K406la suppresses CD8^+^ T‐cell antitumor immune responses predominantly via the HMGCR‐cholesterol core regulatory pathway.

To understand how HADHA K406la–HMGCR–cholesterol regulates signaling pathways in CD8^+^ T cells in GC, we analyzed the RNA‐seq data again (data in Figure 7B). RNA‐seq analysis revealed that blocking HADHA K406la significantly inhibited the expression of endoplasmic reticulum (ER)‐stress‐response genes in CD8^+^ T cells (Figure S11A). Subsequently, qRT‒PCR and flow cytometry revealed that the expression level of Xbp1 in CD8^+^ T cells cocultured with HADHA K406R MCF cells was significantly reduced (Figure S11B,C). When CD8^+^ T cells overexpressed Xbp1, PD‐1 expression significantly increased, and GZMB expression was inhibited (Figure S11D,E). Notably, overexpression of Xbp1 in CD8^+^ T cells suppressed the CD8^+^ T‐cell immune response caused by blocking HADHA K406la (Figure S11D,E). These results indicate that the secondary ER‐stress–XBP1 pathway serves as an auxiliary downstream mechanism activated by the HADHA K406la–HMGCR–cholesterol axis, further contributing to reduced CD8^+^ T‐cell antitumor immune function.

### 3.9 Cell‑Type‑Specific Origin of HMGCR‑Dependent Cholesterol Biosynthesis in the Tumor Microenvironment

Given the potential confounding effect of lactate accumulation downstream of HADHA K406 lactylation, which may activate STAT3 in myeloid cells and modulate T‑cell function via alternative routes, we further clarified the cell‑type‑specific origin of HMGCR‑dependent cholesterol production in the tumor microenvironment. We first examined endogenous HMGCR expression in MDSCs co‑cultured with HADHA WT or K406R gastric cancer cells. Comparable HMGCR levels were observed in MDSCs across different co‑culture groups, indicating that MDSCs do not rely on intrinsic cholesterol biosynthesis but instead sense tumor‑derived exogenous cholesterol (Figure S12A,B). We then performed co‑culture rescue assays using HMGCR‑knockout gastric cancer cells. Ablation of tumor‑cell‑intrinsic HMGCR largely abolished the M2‑like polarization and STAT3 phosphorylation in MDSCs triggered by HADHA K406la, confirming that tumor‑cell‑derived cholesterol, rather than non‑specific lactate‑dependent signaling, constitutes the core driver for MDSC immunosuppressive activation (Figure S12C,D). In parallel, we assessed endogenous HMGCR abundance in tumor‑infiltrating CD8^+^ T cells from HADHA WT and K406R xenograft tumors. No significant difference in HMGCR expression was detected in infiltrating T cells between the two groups, supporting that microenvironmental cholesterol alterations are mainly driven by tumor‑cell‑autonomous metabolic reprogramming, rather than intrinsic cholesterol metabolism within immune cells (Figure S12E,F). Collectively, these data verify that HADHA K406la remodels cholesterol secretion by gastric cancer cells, and the resulting microenvironmental cholesterol pool modulates both MDSC activation and CD8^+^ T‑cell effector function, with lactate‑mediated off‑target effects not representing the dominant mechanism in this regulatory cascade.

### 3.10 Pharmacological Inhibition of Cholesterol Synthesis and Lactate Synthesis Significantly Inhibits GC Progression

Stiripentol, a known lactate dehydrogenase A (LDHA) inhibitor clinically used in anti‐epileptic therapy [74, 75], has also been reported to suppress protein lactylation [76]. To further elucidate the mechanistic relationships among HADHA K406la, cholesterol secretion, and immune suppression, we treated GC cells with the lactate inhibitor stiripentol. Treatment with stiripentol markedly reduced intracellular lactate levels (Figure S13A), HADHA K406la (Figure S13B), and cholesterol secretion (Figure S13C). On the basis of these in vitro findings, we next evaluated the therapeutic potential of combining lactate and cholesterol synthesis inhibitors in a GC model. The mice were treated with simvastatin, stiripentol, or their combination, following the schedule depicted in Figure 9A. Longitudinal body weight monitoring revealed no significant intergroup differences over the treatment period, indicating that all regimens were well tolerated (Figure S13D). In vivo, both single‐agent treatments significantly suppressed tumor growth and improved mouse survival (Figure 9B–D). Notably, compared with monotherapy, the combined administration of simvastatin and stiripentol resulted in more pronounced inhibition of tumor growth and a further survival benefit (Figure 9B–D). We next explored alterations in the tumor immune microenvironment. Stiripentol monotherapy increased the intratumoral infiltration of CD8^+^ T cells and reduced the accumulation of myeloid‐derived suppressor cells (MDSCs) (Figure 9E,F). Importantly, compared with the individual monotherapies, the combination of simvastatin and stiripentol had a more potent regulatory effect on CD8^+^ T cells and MDSCs (Figure 9E,F). To further validate the therapeutic effects of simvastatin and stiripentol in a clinically relevant human model, we established patient‐derived organoids (PDOs) from clinical gastric cancer specimens. Pharmacological intervention with either simvastatin or stiripentol significantly suppressed the growth of gastric cancer organoids. Notably, compared with single‐agent therapy, combined treatment substantially increased the inhibitory effect on organoid growth (Figure 9G), corroborating our in vitro cell and in vivo findings and further supporting the translational potential of cotargeting cholesterol synthesis and lactate production in gastric cancer. Collectively, these findings demonstrate that dual targeting of cholesterol synthesis and lactate production using simvastatin and stiripentol constitutes a promising therapeutic strategy for GC.

**FIGURE 9:**
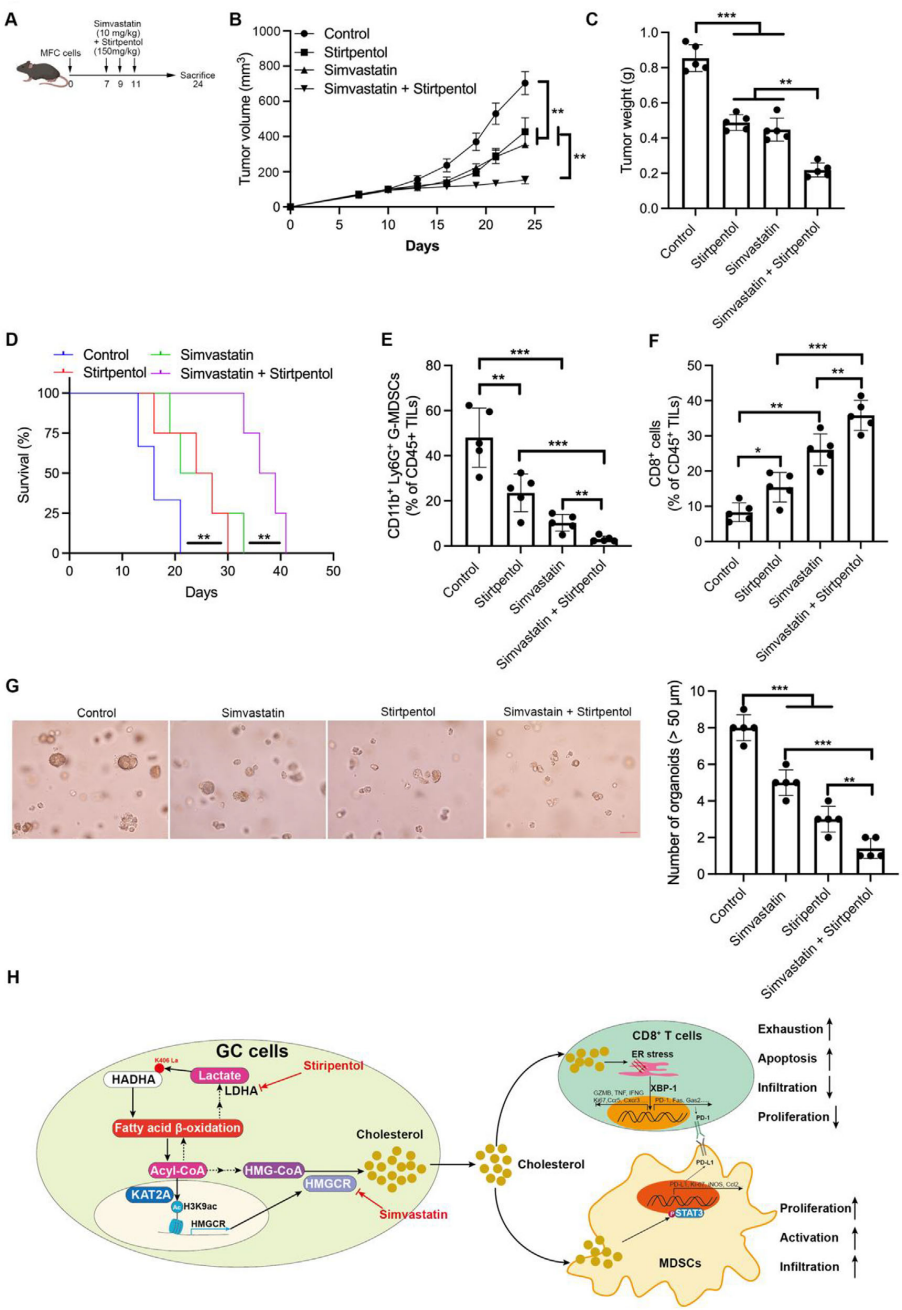
Pharmacological inhibition of cholesterol and lactate synthesis significantly inhibits GC progression. (A) C57BL/6J mice were inoculated subcutaneously with 5 × 10^4^ HADHA ^WT^ or HADHA ^K406R^ MFC cells. On days 7, 9, and 11 after tumor cell inoculation, the mice were intraperitoneally injected with 25 mg/kg simvastatin and 150 mg/kg stiripentol (mean ± SD, n = 5). (B–D) Tumor growth curve (B), tumor weight (C) and survival curve (D) in A (mean ± SD, n = 5). (E, F) Percentages of tumor‐infiltrating CD8^+^ T cells and MDSCs determined by flow cytometry. (G) Morphological observation and statistical quantification of tumor sphere growth in gastric cancer PDOs. PDOs were exposed to vehicle control, 10 µM simvastatin, 20 µM stiripentol, or their combination for 7 days (mean ± SD, n = 5, scale bar = 50 µm). **p < 0.01 and ***p < 0.001.

## 4 Discussion

Enhanced glycolytic flux and consequent lactate build‐up are hallmarks of many malignancies [17]. Emerging evidence indicates that intracellular lactate can give rise to a newly recognized posttranslational modification, lysine lactylation (Kla), on core histones. Nevertheless, how lactylation influences key biological processes in GC remains poorly defined. In this study, we found that HADHA K406la was most significantly upregulated in GC tissues. Lactylation of HADHA^K406^ promotes GC progression by regulating its enzymatic activity and remodeling energy metabolism. In addition, lactylation of HADHA^K406^ shapes the immunosuppressive microenvironment of GC through the HMGCR‐mediated cholesterol synthesis pathway. Finally, the combination of HMGCR inhibitors with lactate dehydrogenase A (LDHA) inhibitors significantly improved the immune response of patients with GC and inhibited GC progression. In summary, we elucidated that HADHA K406la affects GC progression by regulating metabolic pathways and provide a potential therapeutic target for GC treatment (Figure 9H).

Numerous posttranslational modifications (PTMs) have been demonstrated to regulate diverse biological processes within the tumor microenvironment, including the proliferation of malignant cells, immune cell activation, and metabolic reprogramming [77, 78]. These findings suggest that targeting PTMs in cancer cells has significant therapeutic potential. One striking example is lysine lactylation (Kla), a recently characterized posttranslational modification generated from intracellular lactate [79, 80]. Considering the enhancement of glycolysis and lactate accumulation in tumor cells [81], Kla is likely to play important functional roles in malignancies such as GC. As an additional regulatory layer beyond the classical central dogma, Kla may encode substantial, yet underexplored, biological information. To determine its relevance in GC, particularly with respect to nonhistone substrates, we analyzed paired tumor and adjacent gastric tissues from 10 surgically treated patients. Comparative lactylome profiling revealed that compared with their counterparts in adjacent nontumor tissues, individual proteins in tumor samples carried substantially more Kla sites. Furthermore, functional enrichment of differentially modified proteins strongly correlated with fatty acid degradation pathways. Among these proteins, those such as HADHA and ADH1C were prominently upregulated, suggesting a potential connection between Kla and GC development. Deficiency of mitochondrial trifunctional proteins is attributed to impaired fatty acid β‐oxidation (FAO) caused by alterations in hydroxyacyl‐CoA dehydrogenase/3‐ketoacyl‐CoA thiolase/enoyl‐CoA hydratase subunit A (HADHA). Consistent with this finding, HADHA is a critical regulator of energy metabolism and metabolic homeostasis and has been linked to various human diseases, particularly metabolic disorders [82]. More recently, its involvement in cancer biology has attracted increased attention. HADHA has been proposed to exert pro‐tumorigenic effects in lymphoma and lung cancer [83] while acting as a tumor suppressor in clear cell renal cell carcinoma [84], underscoring a context‐dependent role whose molecular mechanisms remain incompletely defined.

In this study, we observed a large number of Kla sites on histones, which is consistent with the findings of previous reports, suggesting that histone lactylation plays a role in epigenetic regulation. However, the subsequent functional experiments in this study focused on Kla modifications on nonhistone proteins. This choice was based on two considerations. First, nonhistone proteins encompass core functional proteins (such as metabolic enzymes and signaling molecules) that drive tumorigenesis, and their modification status may more directly regulate the malignant biological behaviours of GC cells. Second, compared with that of histone Kla, the functional landscape of nonhistone Kla in GC remains largely unexplored, and this study aims to investigate this emerging area. Our results confirmed that the modification of a key metabolic enzyme by HADHA K406la promotes GC cell proliferation by regulating its activity, highlighting the unique and significant role of nonhistone Kla in GC pathogenesis. In the future, in‐depth investigations of the synergistic effects of histone Kla and specific nonhistone Kla modifications will provide a more comprehensive understanding of the lactylation modification network in GC.

Hydroxy‐3‐methylglutaryl coenzyme A reductase (HMGCR) serves as the key rate‐controlling enzyme in the mevalonate pathway, which underpins *de novo* cholesterol production [41, 85, 86]. HMGCR has been reported to be overexpressed in multiple malignancies, including gastric [87], bladder [88], and liver [89] cancers, where it supports tumor cell growth. In this study, we demonstrated that the lactylation of HADHA ^K406^ increases histone acetylation in the HMGCR promoter region and promotes its transcription, thereby upregulating cholesterol levels. In addition, HADHA also generates acetyl‐CoA [90], which is the starting material for cholesterol synthesis [91], through the beta‐oxidation of fatty acids. This may be a more direct manner, in addition to the manner in which the HADHA–HMGCR pathway affects cholesterol synthesis. The overexpression of HMGCR upregulated the cholesterol content and PD‐1 expression in CD8^+^ T cells in HADHA ^K406R^ tumors but inhibited the expression of killer molecules in HADHA ^K406R^ tumors. These results suggested that HADHA K406la promotes T‐cell depletion by promoting HMGCR transcription, thereby promoting GC progression. More importantly, comparative analysis revealed that compared with the controls, the HADHA ^K406R^ GC group had more inflammation‐associated CD8^+^ T cells and a lower proportion of macrophages, suggesting that CD8^+^ T cells may function in the ability of HADHA K406 to promote GC progression.

The HADHA ^K406R^ mutation reduced the ability of HADHA to promote GC development in a nude mouse tumor model, indicating that HADHA K406la plays a T‐cell‐independent role in GC development. HADHA K406la may contribute to the malignant proliferation of GC cells by modulating the abundance of cholesterol within their plasma membranes [92, 93]. Cholesterol secreted by tumors affects the function of CD8^+^ T cells [94]; therefore, we continued to explore whether the cholesterol synthesis pathway mediated by HADHA K406la also affects the infiltration and function of immune cells in GC. Our experimental results confirmed that cholesterol synthesis mediated by HADHA K406la in GC can also affect the infiltration and function of immune cells, leading to the progression of GC.

Although our preclinical data demonstrate a pronounced antitumor and immunomodulatory benefit of combined cholesterol and lactate synthesis inhibition, several pharmacokinetic and safety considerations should be carefully evaluated before clinical translation. Simvastatin is predominantly metabolized by cytochrome P450 3A4 (CYP3A4), whereas stiripentol is a known inhibitor of multiple CYP enzymes, including CYP3A4 and CYP2C19 [95, 96]. Clinical studies in epilepsy have shown that stiripentol can substantially increase systemic exposure to coadministered CYP3A4 substrates, raising concerns about dose‐dependent toxicity [97]. In the context of statins, elevated plasma levels are associated with an increased risk of myopathy and hepatotoxicity, which may be particularly relevant in cancer patients with compromised metabolic reserve [98]. Importantly, recent clinical and translational studies suggest that statins can exert antitumor and immunomodulatory effects at doses lower than those typically used for cardiovascular indications, potentially allowing therapeutic efficacy while minimizing systemic toxicity [99]. Moreover, emerging evidence indicates that lactate metabolism inhibitors may primarily affect the tumor microenvironment, reshaping immune cell function and metabolic competition in situ [100], suggesting that optimized dosing strategies or tumor‐targeted delivery systems could further reduce off‐target effects. Future studies should therefore focus on defining the minimal effective doses, evaluating alternative statins with more favorable pharmacokinetic profiles (e.g., hydrophilic statins), and systematically assessing liver and muscle toxicity in long‐term treatment settings. Collectively, while our findings highlight the therapeutic promise of cotargeting cholesterol and lactate metabolism to enhance antitumor immunity in GC, rigorous pharmacokinetic modeling and safety evaluation will be essential to ensure the safe clinical translation of this combination strategy.

Our in vivo results demonstrated that single or combined treatment with simvastatin and stiripentol achieved favorable safety without causing obvious body weight loss in tumor‐bearing mice, confirming the good in vivo tolerability of both the monotherapy and combined regimens. Moreover, cotargeting cholesterol synthesis and lactate production strongly suppressed GC growth and prolonged survival, accompanied by remodeling of the tumor immune microenvironment. Specifically, stiripentol alone increased intratumoral CD8^+^ T‐cell infiltration and reduced MDSC accumulation, whereas the combination of simvastatin and stiripentol resulted in a more potent immune‐modulatory effect. Given the evident improvement in the abundance of cytotoxic CD8^+^ T cells and the alleviation of immunosuppressive MDSCs, this metabolic intervention is mechanistically poised to reverse T‐cell exhaustion and may exert synergistic antitumor effects with immune checkpoint blockade. Therefore, future work will further explore the therapeutic efficacy and synergistic mechanism of simvastatin plus stiripentol combined with anti‐PD‐1/PD‐L1 immunotherapy in preclinical GC models, which will provide more translational evidence to facilitate the clinical application of this combined metabolic and immune therapeutic strategy.

In summary, we elucidate a previously unrecognized mechanism by which the tumor microenvironment drives functional exhaustion of CD8^+^ T cells. Specifically, HADHA K406la enhances HMGCR transcription, thereby increasing cholesterol metabolism and facilitating GC progression. These findings indicate that the modulation of cholesterol homeostasis may represent a promising approach to augment the antitumor efficacy of CD8^+^ T‐cell‐based immunotherapy. Our work further highlights the importance of tightly regulating lipid metabolism in CD8^+^ T cells and/or the tumor microenvironment to improve clinical responses to cancer immunotherapy. Moreover, our data support a model in which tumor‐derived cholesterol contributes to the dysfunction of tumor‐infiltrating CD8^+^ T cells. Nonetheless, the broader functional roles of HADHA K406la in cholesterol metabolism and T‐cell biology require more in‐depth investigation, and the precise molecular and structural mechanisms by which HADHA lactylation upregulates HMGCR need to be clarified in future studies.

## Author Contributions

M.F., Q.J., J.Z., and Y.R. performed the core experiments, carried out data analyses, and drafted the manuscript. J.T., X.S., Z.L., Y.C., and X.L. contributed to the establishment of the animal models. Y.W. and G.Z. conceived and supervised the study and critically revised the manuscript.

## Study Registration

This study was a retrospective observational cohort study and did not involve prospective patient interventions. It was not registered in a clinical trial registry because it does not fulfil the ICMJE definition of a clinical trial.

## Conflicts of Interest

The authors declare no conflicts of Interest.

## Supporting information




**Supporting File**: advs78291‐sup‐0001‐SuppMat.docx.

## Data Availability

The data that support the findings of this study are openly available in Gene Expression Omnibus at https://www.ncbi.nlm.nih.gov/geo/query/acc.cgi?acc=GSE276927, reference number GSE276927.
