# Supplementary material for: HADHA K406 Lactylation Regulates Metabolic Reprogramming and Suppresses Immune Responses in Gastric Cancer
Source: Adv Sci (Weinh). 2026 Oct 29:e78291. Online ahead of print. doi: 10.1002/advs.78291 (PMC13720267; doi:10.1002/advs.78291)
Supplement: Supplementary file 1 [file ADVS-9999-e78291-s001.docx]

**Supplementary figure and table**

**Supplementary Figure 1. Overview of the research workflow and quality control for mass spectrometry-based lactylome profiling.** (A) Schematic overview of the 10 gastric cancer patients enrolled for individualized multi-omics profiling, with detailed clinical information summarized. (B) Principal component analysis (PCA) of the global proteome dataset. (C) Distribution of the mass-to-charge (m/z) ratio and peptide length for differentially charged peptides. (D, E) Quality control evaluation based on Spearman correlation coefficients (D) and relative standard deviation (RSD) values (E) across biological replicates of the lactylome data. (F) Box plots showing the overall protein identification distribution in gastric tumor samples. (G) Statistical distribution of lysine lactylation (Kla) sites per protein in tumor tissues and paired adjacent normal tissues. (H) Motif-X analysis illustrating the top ten enriched upstream and downstream amino acids flanking the central lactylated lysine residue. (I) Forest plot of multivariate Cox regression analysis evaluating the independent prognostic significance of clinical parameters and lactylation-related indicators. HR, hazard ratio; CI, confidence interval. (J) Functional enrichment analysis of identified lactylated proteins.

**Supplementary Figure 2. Elevated HADHA K406 lactylation in gastric cancer cells.** (A) Representative mass spectrum of the peptide containing HADHA K350 lactylation. (B) LC-MS/MS identification of endogenous HADHA lactylation sites in NCI-N87 cells. (C–E) Dot blot assays validating the efficiency and specificity of custom anti-HADHA-K406la and anti-HADHA-K350la site-specific antibodies. (F) Western blot analysis of total HADHA and K350-lactylated HADHA levels in multiple gastric cancer cell lines (SNU-1, HGC-27, KATO-III, NCI-N87, MFC) and the normal gastric epithelial cell line GES-1. (G) Quantification of intracellular lactate concentrations in gastric cell lines (GES-1, SNU-1, HGC-27, KATO-III, NCI-N87, MFC). Data are presented as mM (mean ± SD, n = 5). (H, I) Generation of HADHA-knockout gastric cancer cell lines (H), followed by stable reconstitution with wild-type (WT) HADHA or the lactylation-deficient HADHA K406R mutant (I). (J) Co-immunoprecipitation assay verifying that the K406R mutation efficiently abolishes HADHA K406 lactylation. (K) Structural prediction of WT HADHA and HADHA K406R mutant using AlphaFold2. (L) Structural alignment of WT and mutant HADHA proteins performed in PyMOL. (M) Schematic illustration of HADHA functional domains (UniProt ID: P40939; https://www.ebi.ac.uk/interpro/protein/UniProt/P40939/). (N, O) Validation of HADHA K406 lactylation in a cell‑free system via Western blot (N) and mass spectrometry (O). (P) Western blot detection of HADHA K406 lactylation levels following CRISPR‑Cas9-mediated knockout of potential delactylases. **p < 0.01, ***p< 0.001, ****p < 0.0001.

**Supplementary Figure 3. HADHA K406 lactylation promotes malignant progression of gastric cancer cells.** (A, B) Measurement of cellular oxidative phosphorylation capacity via oxygen consumption rate (OCR) in HADHA-knockout MFC cells reconstituted with HADHA ^WT^ or HADHA ^K406R^ (mean ± SD, n = 4). (C) Quantification of intracellular and extracellular lactate levels in the above cell groups (mean ± SD, n = 4). (D–I) Functional assays evaluating cell proliferation, migration, and invasion capacities in NCI-N87 and MFC HADHA-knockout cells expressing HADHA ^WT^ or HADHA ^K406R^ (mean ± SD, n = 3, scale bar = 50 μm). (J–O) Subcutaneous xenograft tumor models established by injecting HADHA WT or HADHA K406R-overexpressing MFC and NCI-N87 cells into nude mice (n = 5). Representative tumor images (J, M), tumor growth curves (K, N), and tumor weight statistics (L, O) are shown (mean ± SD, scale bar = 1 cm). *p < 0.05, **p < 0.01, ***p < 0.001.

**Supplementary Figure 4. Functional validation of HADHA K406 lactylation in gastric cancer progression and metabolic reprogramming.** (A) Western blot validation of WT HADHA, lactylation-mimetic K406Q, and lactylation-deficient K406R mutant expression in HADHA-knockout gastric cancer cells. (B–G) Assessment of HADHA enzymatic activity (B), cellular acetyl-CoA content (C), and malignant phenotypes including cell proliferation and invasion (D–G) in cells expressing HADHA WT, K406Q, or K406R. K406Q mimics constitutive lactylation, whereas K406R mimics a permanently non-lactylated state (mean ± SD, n = 4). (H) Western blot analysis of HADHA K406 lactylation in WT and K406R cells treated with the LDH inhibitor FX11 (5 μM) or vehicle control for 48 h. (I–K) Detection of HADHA enzymatic activity (I), acetyl-CoA levels (J), and glycolytic capacity and glycolytic reserve (K) in HADHA WT cells after FX11 intervention (mean ± SD, n = 4). ***p** < 0.05, ****p** < 0.01, *****p** < 0.001; n.s., not significant.

**Supplementary Figure 5. HADHA K406 lactylation upregulates HMGCR and cholesterol synthesis via KAT2A-mediated histone acetylation and the RIG-I/AMPKα/CRTC2/SREBP-2 axis.** (A) Transcriptomic comparison of the expression profiles of major histone acetyltransferases in HADHA ^WT^ versus HADHA ^K406R^ cells. (B) Immunoblot analysis of alternative H3K9 modifications, including methylation, lactylation, and butyrylation, between HADHA ^WT^ and HADHA ^K406R^ cells. (C) CRISPR screening results showing the effects of individual histone acetyltransferase knockout on HMGCR expression. (D) Western blot detection of H3K9ac and HMGCR protein levels in HADHA ^WT^ and HADHA ^K406R^ cells with or without KAT2A knockdown. (E) Detection of intracellular cholesterol synthesis levels in HADHA ^WT^ and HADHA ^K406R^ cells upon KAT2A depletion. (F) ChIP-seq analysis of KAT2A chromatin binding profiles in HADHA ^WT^ and HADHA ^K406R^ cells. (G) IGV visualization of differential KAT2A enrichment at the *Hmgcr* promoter in HADHA ^WT^ versus HADHA ^K406R^ cells. (H) ChIP-qPCR validation of KAT2A binding efficiency at the *Hmgcr* promoter region (mean ± SD, n = 4). (I) Western blot analysis of RIG-I and phosphorylated AMPKα expression in NCI-N87 cells expressing HADHA WT or K406R. (J) Co-immunoprecipitation assay detecting the endogenous interaction between RIG-I and AMPKα in the above cell groups. (K, L) qRT-PCR and Western blot analyses of CRTC2 and SREBP-2 mRNA and protein expression in HADHA ^WT^ and HADHA ^K406R^ cells (mean ± SD, n = 3). *p < 0.05, **p < 0.01; n.s., not significant.

**Supplementary Figure 6. Single-cell transcriptomic profiling of HADHA WT and HADHA K406R xenograft tumors.** (A) Schematic workflow of single-cell RNA-seq analysis. (B) UMAP visualization of nine distinct cell clusters identified in tumor tissues. (C, D) Heatmap of canonical marker genes for each cell subtype (C) and functional enrichment analysis of distinct cell populations (D). (E, F) Comparative distribution (E) and proportion (F) of each cell subtype in HADHA ^WT^ and HADHA ^K406R^ tumors.

**Supplementary Figure 7. Immune cell landscape in HADHA ^WT^ and HADHA ^K406R^ tumors.** (A) UMAP plots showing the nine defined cell populations in individual HADHA ^WT^ and HADHA ^K406R^ tumor samples. (B) Statistical proportions of each cell subtype in different tumor groups. (C–G) Module score analysis of gene signatures related to cholesterol uptake, synthesis, efflux, storage, and overall cholesterol function in tumor-infiltrating CD8⁺ T cells from HADHA ^WT^ and HADHA ^K406R^ tumors. (H–L) Bubble plots illustrating the expression patterns of gene sets associated with cytokine secretion, cytotoxicity, exhaustion-related transcription factors, early T cell activation, and T cell exhaustion in CD8⁺ T cells from HADHA ^WT^ and HADHA ^K406R^ tumors.

**Supplementary Figure 8.** **HADHA ^K406R^ regulates the functions of CD8^+^ T cells and myeloid cells in GC.** (A) Western blotting analysis of HADHA K406 lactylation levels in fresh human gastric cancer tissues. (B, C) Flow cytometric analysis of PD-1, IFN-γ and TNF expression in CD8⁺ T cells from groups with high or low HADHA K406 lactylation (mean ± SD, n = 4). (D, E) CD8^+^ T cells isolated from C57BL/6J mouse were cultured in RPMI 1,640 medium with CD3/CD28 antibodies in the presence of IL-2. On day 3, cells were treated with cholesterol for 3 days (D) or 5 mM β-CD for 24 h (E). The percentage of PD-1^+^ CD8^+^ T cells was determined by flow cytometry (mean ± SD, n = 5). (F, G) C57BL/6J mice were inoculated subcutaneously with 5 x 10^4^ HADHA ^WT^ or HADHA ^K406R^ MFC cells. On days 7, 9, and 11 after tumor cells inoculation, mice were intraperitoneally injected with 25 mg/kg simvastatin (mean ± SD, n = 5). (H-J) The percentage of tumor-infiltrating GZMB^+^ CD8^+^ T cells (H, I) and PD-1^+^ T cells (J) of total tumor-infiltrating leukocytes in tumors were analyzed by flow cytometry (mean ± SD, n = 5). *P <0.05, **P <0.01 and ***P <0.001.

**Supplementary Figure 9. HADHA K406 lactylation activates MDSCs via the HMGCR-cholesterol-STAT3 signaling axis.** (A) Western blot analysis of total and phosphorylated STAT3 levels in MDSCs co-cultured with HADHA WT or K406R-overexpressing GC cells. (B) qRT-PCR detection of *iNOS* mRNA expression in co-cultured MDSCs (mean ± SD, n = 4). (C) Flow cytometric measurement of ROS levels in MDSCs after co-culture with different tumor cell groups (mean ± SD, n = 4). (D) Flow cytometric analysis of GZMB expression in CD8⁺ T cells within the tumor–MDSC co-culture system (mean ± SD, n = 4). (E, F) Flow cytometric detection of CD206 levels in MDSCs with STAT3 or iNOS knockdown after co-culture with HADHA ^WT^ or HADHA ^K406R^ tumor cells (mean ± SD, n = 4). (G) Flow cytometry quantification of M2-type macrophage polarization in HADHA ^WT^ and HADHA ^K406R^ tumor tissues (mean ± SD, n = 5). **p < 0.01.

**Supplementary Figure 10. HMGCR-driven cholesterol accumulation induces CD8⁺ T cell exhaustion.** (A) Western blot validation of HMGCR knockdown and overexpression efficiency in MFC gastric cancer cells. (B) Quantification of intracellular cholesterol content in HMGCR-manipulated cell lines (mean ± SD, n = 5). (C, D) Phenotypic analysis of CD8⁺ T cells cultured with tumor supernatant derived from shHMGCR or control cells. The proportions of PD-1⁺, GZMB⁺, and IFN-γ⁺ CD8⁺ T cells were determined by flow cytometry (mean ± SD, n = 5). (E, F) Functional detection of CD8⁺ T cells cultured with supernatant from HMGCR-overexpressing or control tumor cells (mean ± SD, n = 5). (G) Flow cytometric analysis of PD-1 expression in CD8⁺ T cells directly treated with purified exogenous cholesterol (mean ± SD, n = 5). (H, I) *In vivo* flow cytometry analysis of GZMB and IFN-γ expression in tumor-infiltrating CD8⁺ T cells from shHMGCR (H) and HMGCR-overexpressing (I) xenograft models (mean ± SD). (J) Flow cytometric detection of GZMB and IFN-γ levels in tumor-infiltrating CD8⁺ T cells derived from mice bearing HMGCR-overexpressing HADHA ^WT^ or HADHA ^K406R^ tumors (mean ± SD, n = 5). **p < 0.01, ***p < 0.001, ****p < 0.0001.

**Supplementary Figure 11. HADHA K406 lactylation impairs CD8⁺ T cell antitumor immunity via HMGCR-cholesterol-mediated XBP1 activation.** (A) Heatmap showing differential expression of endoplasmic reticulum (ER) stress-related genes in CD8⁺ T cells co-cultured with HADHA ^WT^ or HADHA ^K406R^ tumor cells, as determined by RNA-seq. (B, C) Validation of XBP1 expression in co-cultured CD8⁺ T cells via qRT-PCR (B) and flow cytometry (C) (mean ± SD, n = 4). (D, E) Flow cytometric detection of GZMB and PD-1 expression in XBP1-overexpressing CD8⁺ T cells after co-culture with HADHA ^WT^ or HADHA ^K406R^ tumor cells (mean ± SD, n = 4). ****p** < 0.01, *****p** < 0.001.

**Supplementary Figure 12. Tumor‑cell‑intrinsic HADHA K406la mutation does not affect HMGCR expression in macrophages and CD8⁺ T cells.** (A, B) Co‑culture of MFC cells bearing HADHA‑WT or HADHA K406R with mouse bone‑derived macrophages (BMDMs). HMGCR expression levels in BMDMs were determined by RT‑qPCR (A) and western blot (B). (C) Flow‑cytometric analysis of M2 polarization of BMDMs under the above co‑culture conditions. (D) Western blot analysis of p‑STAT3 expression in BMDMs from the above co‑culture system. (E, F) RT‑qPCR (E) and western blot (F) analysis of HMGCR expression in CD8⁺ T cells isolated from HADHA‑WT and HADHA K406R tumors. ns, not significant; **p < 0.01.

**Supplementary Figure 13. Pharmacological inhibition of lactate production decreases HADHA K406 lactylation and cholesterol secretion in gastric cancer cells.** (A) Quantification of intracellular lactate levels in gastric cancer cells after Stiripentol treatment (mean ± SD, n = 4). (B) Western blot analysis of HADHA K406 lactylation levels upon Stiripentol intervention. (C) Detection of secreted cholesterol levels in control and Stiripentol-treated groups (mean ± SD, n = 4). (D) Mouse body weight monitoring during in vivo treatment. No significant differences were observed among groups by two-way ANOVA (main effect, p = 0.5419; interaction effect, p = 0.3825; n = 5). *****p** < 0.001.

**Supplementary table 1**

| **Characteristics and clinical data of GC patients, related to Figure 1G.** | | | | | | | | |
| --- | --- | --- | --- | --- | --- | --- | --- | --- |
| **Sample.ID** | **Participant** | **Type** | **Gender** | **Age** | **Histology Type** | **Primary Tumor Location** | **TNM Classification** | **Staging** |
| **GC01T** | **GC01** | **Tumor** | **female** | **67** | **Adenocarcinoma** | **Antrum, Pylorus** | **T2N3aM0** | **IIIA** |
| **GC01A** | **GC01** | **Adjacent tissue** | **female** | **67** | **/** |  |  |  |
| **GC02T** | **GC02** | **Tumor** | **female** | **65** | **Adenocarcinoma** | **Antrum** | **T2N1M0** | **IIA** |
| **GC02A** | **GC02** | **Adjacent tissue** | **female** | **65** | **/** |  |  |  |
| **GC03T** | **GC03** | **Tumor** | **male** | **53** | **Adenocarcinoma** | **Lower body** | **T4aN1M0** | **IIIA** |
| **GC03A** | **GC03** | **Adjacent tissue** | **male** | **53** | **/** |  |  |  |
| **GC04T** | **GC04** | **Tumor** | **male** | **51** | **Adenocarcinoma with partial signet ring cell carcinoma** | **Lower body** | **T3N3aM0** | **IIIB** |
| **GC04A** | **GC04** | **Adjacent tissue** | **male** | **51** | **/** |  |  |  |
| **GC05T** | **GC05** | **Tumor** | **male** | **59** | **Adenocarcinoma** | **Antrum** | **T1bN2M0** | **IIA** |
| **GC05A** | **GC05** | **Adjacent tissue** | **male** | **59** | **/** |  |  |  |
| **GC06T** | **GC06** | **Tumor** | **female** | **65** | **Adenocarcinoma** | **Antrum** | **T4aN3aM0** | **IIIB** |
| **GC06A** | **GC06** | **Adjacent tissue** | **female** | **65** | **/** |  |  |  |
| **GC07T** | **GC07** | **Tumor** | **female** | **54** | **Adenocarcinoma with partial signet ring cell carcinoma** | **Cardia** | **T3N1M0** | **IIB** |
| **GC07A** | **GC07** | **Adjacent tissue** | **female** | **54** | **/** |  |  |  |
| **GC08T** | **GC08** | **Tumor** | **female** | **59** | **Adenocarcinoma with partial signet ring cell carcinoma** | **Antrum** | **T1bN0M0** | **IA** |
| **GC08A** | **GC08** | **Adjacent tissue** | **female** | **59** | **/** |  |  |  |
| **GC09T** | **GC09** | **Tumor** | **female** | **66** | **Adenocarcinoma** | **Lower body** | **T4aN1M0** | **IIIA** |
| **GC09A** | **GC09** | **Adjacent tissue** | **female** | **66** | **/** |  |  |  |
| **GC10T** | **GC10** | **Tumor** | **male** | **61** | **Adenocarcinoma** | **Cardia** | **T3N3aM0** | **IIIB** |
| **GC10A** | **GC10** | **Adjacent tissue** | **male** | **61** | **/** |  |  |  |

**Supplementary table 2**

| Location | Sex | Age | Path_Type | Grade | Surgery | T | N | M | Stage | Part | Size | Borr | Cancer_embolus | Nerve_Invasion | Surv_Time | Surv_Status | Total_la_group | HADHAK406la_group |
| --- | --- | --- | --- | --- | --- | --- | --- | --- | --- | --- | --- | --- | --- | --- | --- | --- | --- | --- |
| C5 | Male | 54 | Adenocarcinoma | II | PG | 2 | 2 | 0 | 2b |  | 1 |  | No | No | 0.5 | 1 | High | High |
| D15 | Female | 77 | Adenocarcinoma | II-III | DG | 4a | 3a | 0 | 3c | Antrum | 3 | Infiltrative_ulcerative | Yes | Yes | 0.5 | 1 | High | High |
| F16 | Male | 60 | Adenocarcinoma | III | PG | 4a | 0 | 0 | 2b | Body | 4 | Infiltrative_ulcerative |  | No | 1 | 1 | High | High |
| I10 | Female | 45 | Signet_ring_cell_carcinoma |  | DG | 4a | 2 | 0 | 3b | Lesser_curvature | 5 | Infiltrative_ulcerative | No | No | 1 | 1 | High | High |
| J2 | Female | 51 | Poorly_diff_adeno_with_signet |  |  | 4a | 3a | 0 | 3c | Greater_curvature | 6 | Infiltrative_ulcerative | No | No | 1 | 1 | High | High |
| C4 | Male | 65 | Adenocarcinoma | II | DG | 2 | 1 | 0 | 2a | Antrum | 4 | Infiltrative_ulcerative | No | No | 2 | 1 | High | High |
| C10 | Male | 69 | Adenocarcinoma | II | DG | 4a | 2 | 0 | 3b | Antrum | 6 | Ulcerative | No | No | 3 | 1 | High | High |
| I12 | Female | 62 | Signet_ring_cell_carcinoma |  | DG | 3 | 3a | 0 | 3b | Body | 12 | Diffuse_infiltrative | Yes | No | 3 | 1 | High | High |
| J4 | Female | 60 | Signet_ring_cell_carcinoma |  | TG | 4a | 3a | 0 | 3c | Antrum | 8 | Infiltrative_ulcerative | Yes | Yes | 3.5 | 1 | High | High |
| I16 | Male | 88 | Poorly_diff_adeno_with_signet |  | DG | 3 | 2 | 0 | 3a | Antrum | 8 | Infiltrative_ulcerative | No | Yes | 4 | 1 | High | High |
| J5 | Male | 57 | Signet_ring_cell_carcinoma |  |  | 3 | 1 | 0 | 2b |  | 1 | Protuberant | No | No | 4 | 1 | High | High |
| G10 | Male | 54 | Adenocarcinoma | III | TG | 3 | 3a | 0 | 3b | Cardia | 3 | Infiltrative_ulcerative | No | No | 6 | 0 | High | High |
| I8 | Male | 54 | Signet_ring_cell_carcinoma |  | DG | 4a | 3a | 0 | 3c | Antrum | 4 | Infiltrative_ulcerative | No | No | 6.5 | 1 | High | High |
| J9 | Male | 62 | Poorly_diff_adeno_with_signet |  | DG | 4a | 3b | 0 | 3c | Lesser_curvature | 4 | Infiltrative_ulcerative | No | No | 7 | 0 | High | High |
| J12 | Male | 75 | Signet_ring_cell_carcinoma |  | PG | 3 | 0 | 0 | 2a | Cardia | 4 | Ulcerative | No | No | 7.5 | 1 | High | High |
| C7 | Male | 73 | Adenocarcinoma | II | PG | 4a | 1 | 0 | 3a | Cardia | 5 | Infiltrative_ulcerative | Yes | Yes | 7.5 | 1 | High | High |
| F11 | Male | 62 | Poorly_diff_adeno_with_signet | III | DG | 4a | 3b | 1 | 4 | Lesser_curvature | 6 | Ulcerative |  | No | 8 | 1 | High | High |
| F10 | Male | 53 | Adenocarcinoma | III | DG | 1a | 0 | 0 | 2b | Antrum | 7 | Infiltrative_ulcerative |  | No | 10 | 1 | High | High |
| H12 | Female | 49 | Signet_ring_cell_carcinoma |  | TG | 4a | 2 | 0 | 3b | Body | 18 | Diffuse_infiltrative | No | No | 11 | 1 | High | High |
| A3 | Female | 76 | Adenocarcinoma | I-II | TG | 3 | 0 | 0 | 2a | Antrum | 14 | Diffuse_infiltrative | No | No | 12 | 1 | High | High |
| G2 | Male | 59 | Adenocarcinoma | III | TG | 4a | 1 | 0 | 3a | Lesser_curvature | 2 | Ulcerative |  | No | 45 | 1 | High | High |
| H7 | Female | 40 | Adenocarcinoma | III | DG | 2 | 0 | 0 | 1b | Antrum | 6.5 | Infiltrative_ulcerative | No | No | 50 | 1 | High | High |
| E5 | Male | 51 | Adenocarcinoma | II-III | TG | 3 | 0 | 0 | 2a | Lesser_curvature | 8 | Infiltrative_ulcerative | No | No | 80 | 0 | High | High |
| A2 | Male | 72 | Adenocarcinoma | I-II | DG | 1b | 1 | 0 | 3a | Antrum | 8 | Diffuse_infiltrative | No | No | 90 | 0 | High | High |
| E11 | Male | 37 | Adenocarcinoma | II-III | DG | 1b | 1 | 0 | 1 | Antrum | 2 |  | No | No | 1.5 | 1 | Low | High |
| J13 | Female | 54 | Poorly_diff_adeno_with_signet |  | PG | 2 | 0 | 0 | 1b | Cardia | 1 | Ulcerative | No | No | 4 | 1 | Low | High |
| H6 | Female | 52 | Adenocarcinoma | III | DG | 1b | 0 | 0 | 1a | Antrum | 1.8 |  | Yes | No | 7 | 0 | Low | High |
| J8 | Female | 52 | Poorly_diff_adeno_with_signet |  |  | 3 | 2 | 0 | 3a | Antrum | 2.5 | Ulcerative | No | No | 10 | 1 | Low | High |
| G14 | Female | 55 | Adenocarcinoma | III | DG | 2 | 1 | 0 | 2a | Lesser_curvature | 1 | Infiltrative_ulcerative | No | No | 12 | 1 | Low | High |
| E1 | Male | 73 | Adenocarcinoma | II-III | DG | 3 | 2 | 0 | 3a | Lesser_curvature | 3 | Infiltrative_ulcerative | No | No | 14 | 1 | Low | High |
| G12 | Male | 55 | Adenocarcinoma | III | DG | 2 | 0 | 0 | 1b | Lesser_curvature | 2 | Infiltrative_ulcerative | No | No | 15 | 1 | Low | High |
| B11 | Male | 64 | Adenocarcinoma | II | DG | 2 | 0 | 0 | 1b | Antrum | 3.5 | Infiltrative_ulcerative | No | No | 16.5 | 1 | Low | High |
| D13 | Male | 71 | Adenocarcinoma | II-III | DG | 3 | 0 | 0 | 2a | Antrum | 2 | Ulcerative | No | No | 20 | 1 | Low | High |
| G6 | Male | 77 | Poorly_diff_adeno_with_signet | III | DG | 2 | 0 | 0 | 1b | Antrum | 5 | Infiltrative_ulcerative | No | No | 25 | 1 | Low | High |
| D6 | Male | 57 | Adenocarcinoma | II-III | DG | 2 | 1 | 0 | 2a | Lesser_curvature | 4 | Infiltrative_ulcerative | No | No | 38 | 0 | Low | High |
| B6 | Male | 69 | Adenocarcinoma | II | DG | 2 | 0 | 0 | 1b | Lesser_curvature | 4 | Infiltrative_ulcerative | No | No | 42 | 1 | Low | High |
| D3 | Female | 79 | Adenocarcinoma | II-III | DG | 2 | 1 | 0 | 2a | Antrum | 2 | Ulcerative | No | No | 45 | 1 | Low | High |
| B1 | Female | 57 | Adenocarcinoma | II | DG | 2 | 1 | 0 | 2a | Body | 10 | Diffuse_infiltrative |  | No | 63 | 1 | Low | High |
| I2 | Male | 47 | Signet_ring_cell_carcinoma |  | DG | 2 | 0 | 0 | 2b | Antrum | 1 | Infiltrative_ulcerative |  | No | 74 | 1 | Low | High |
| A11 | Female | 52 | Adenocarcinoma | II | TG | 4a | 2 | 0 | 3b | Lesser_curvature | 7 | Infiltrative_ulcerative | No | No | 98 | 1 | Low | High |
| D7 | Male | 69 | Adenocarcinoma | II-III | TG | 4a | 2 | 0 | 3b | Lesser_curvature | 6 | Protuberant | Yes | Yes | 4 | 0 | High | Low |
| H1 | Female | 73 | Adenocarcinoma | III | DG | 3 | 2 | 0 | 3a | Antrum | 3 | Infiltrative_ulcerative | No | No | 6.5 | 1 | High | Low |
| B7 | Female | 74 | Adenocarcinoma | II | DG | 4a | 3a | 0 | 3c | Antrum | 9 | Protuberant | Yes | No | 8 | 0 | High | Low |
| D2 | Male | 54 | Adenocarcinoma | II-III | DG | 4a | 3a | 1 | 4 | Antrum | 3 | Infiltrative_ulcerative |  | No | 10 | 1 | High | Low |
| C12 | Male | 70 | Adenocarcinoma | II-III | DG | 4a | 2 | 1 | 4 | Antrum | 6 | Infiltrative_ulcerative | No | No | 13 | 0 | High | Low |
| H15 | Male | 53 | Signet_ring_cell_carcinoma |  | TG | 4a | 2 | 0 | 3b | Cardia | 4 | Infiltrative_ulcerative |  | No | 16 | 0 | High | Low |
| I5 | Female | 50 | Poorly_diff_adeno_with_signet |  | TG | 4a | 3b | 0 | 3c | Antrum | 12 | Diffuse_infiltrative | Yes | No | 18 | 0 | High | Low |
| E14 | Male | 43 | Adenocarcinoma | III | DG | 4a | 2 | 0 | 3b | Antrum | 0 | Ulcerative | No | No | 21 | 0 | High | Low |
| B13 | Male | 57 | Adenocarcinoma | II | TG | 4a | 2 | 0 | 3b | Remnant_stomach | 5 | Protuberant | No | Yes | 25 | 1 | High | Low |
| G15 | Male | 56 | Adenocarcinoma | III |  | 3 | 2 | 0 | 3a | Body | 13 | Diffuse_infiltrative | No | No | 25 | 1 | High | Low |
| D11 | Female | 40 | Adenocarcinoma | II-III | DG | 2 | 1 | 0 | 2a | Antrum | 3 | Infiltrative_ulcerative | No | No | 30 | 1 | High | Low |
| F5 | Male | 61 | Adenocarcinoma | III | DG | 4a | 1 | 0 | 3a | Body | 16 | Protuberant | Yes | No | 35 | 1 | High | Low |
| H4 | Male | 70 | Adenocarcinoma | III | DG | 2 | 0 | 0 | 1b | Lesser_curvature | 2.7 | Infiltrative_ulcerative | No | No | 60 | 1 | High | Low |
| I4 | Female | 65 | Signet_ring_with_mucin |  | DG | 4a | 3b | 1 | 4 | Antrum | 8 | Infiltrative_ulcerative |  | No | 65 | 1 | High | Low |
| E15 | Male | 46 | Poorly_diff_adeno_with_signet | III | DG | 2 | 2 | 0 | 3a | Lesser_curvature | 5 | Diffuse_infiltrative | No | No | 90 | 1 | High | Low |
| E3 | Male | 73 | Adenocarcinoma | II-III | DG | 3 | 2 | 0 | 3a | Antrum | 4.5 | Infiltrative_ulcerative | No | No | 90 | 0 | High | Low |
| A6 | Male | 60 | Adenocarcinoma | I-II |  | 1b | 0 | 0 | 1 | Antrum | 0 |  | No | No | 1 | 1 | Low | Low |
| B16 | Male | 65 | Adenocarcinoma | II | DG | 4a | 2 | 0 | 3b | Lesser_curvature | 3 | Ulcerative | No | No | 2 | 0 | Low | Low |
| G7 | Male | 62 | Adenocarcinoma | III | TG | 3 | 2 | 0 | 3a | Cardia | 5 | Infiltrative_ulcerative | No | No | 2.5 | 0 | Low | Low |
| I14 | Female | 60 | Signet_ring_cell_carcinoma |  | DG | 1b | 0 | 0 | 1a | Lesser_curvature | 1 |  | No | No | 2.5 | 1 | Low | Low |
| A15 | Female | 74 | Adenocarcinoma | II | DG | 4a | 2 | 0 | 3b | Antrum | 5 | Ulcerative |  | No | 3 | 1 | Low | Low |
| A9 | Male | 54 | Adenocarcinoma | II | DG | 4a | 1 | 0 | 3a | Antrum | 4 | Infiltrative_ulcerative | No | No | 3 | 1 | Low | Low |
| J15 | Male | 38 | Adenocarcinoma |  |  | 1a | 0 | 0 | 1 | Antrum | 1 |  | No | No | 4.5 | 0 | Low | Low |
| E9 | Male | 59 | Adenocarcinoma | II-III |  | 1b | 0 | 0 | 1a | Antrum | 3 |  | No | No | 6 | 0 | Low | Low |
| H9 | Male | 72 | Adenocarcinoma | III | PG | 3 | 1 | 0 | 2b | Cardia | 2.5 | Infiltrative_ulcerative | No | No | 8 | 1 | Low | Low |
| E7 | Male | 67 | Adenocarcinoma | II-III | DG | 2 | 0 | 0 | 1b | Antrum | 4.5 | Infiltrative_ulcerative | No | No | 10.5 | 1 | Low | Low |
| C2 | Male | 60 | Adenocarcinoma | II | DG | 2 | 0 | 0 | 1b | Lesser_curvature | 2 | Infiltrative_ulcerative | No | No | 13 | 1 | Low | Low |
| B9 | Female | 54 | Adenocarcinoma | II | TG | 4a | 0 | 0 | 2b | Cardia | 7 | Infiltrative_ulcerative | No | No | 30 | 0 | Low | Low |
| D9 | Male | 73 | Adenocarcinoma | II-III | DG | 4a | 0 | 0 | 2b | Antrum | 4 | Infiltrative_ulcerative | No | No | 33 | 0 | Low | Low |
| G3 | Male | 49 | Adenocarcinoma | III | DG | 4a | 3a | 0 | 3c | Lesser_curvature | 4 | Infiltrative_ulcerative |  | Yes | 52 | 0 | Low | Low |
| B4 | Male | 52 | Adenocarcinoma | II | DG | 4a | 0 | 0 | 2b | Antrum | 3 | Infiltrative_ulcerative |  | No | 58 | 0 | Low | Low |
| F13 | Female | 49 | Poorly_diff_adeno_with_signet | III | DG | 1b | 0 | 0 | 1a | Antrum | 3 |  |  | No | 89 | 0 | Low | Low |
| F7 | Male | 52 | Poorly_diff_adeno_with_signet | III | DG | 4a | 2 | 0 | 3b | Antrum | 3 | Ulcerative |  | No | 90 | 0 | Low | Low |
| A13 | Female | 60 | Adenocarcinoma | II | DG | 4a | 1 | 0 | 3a | Antrum | 4 | Infiltrative_ulcerative |  | No | 95 | 0 | Low | Low |
| F3 | Male | 60 | Adenocarcinoma | III | DG | 4a | 0 | 0 | 2b | Lesser_curvature | 3 | Ulcerative | No | No | 102 | 0 | Low | Low |
| C15 | Male | 43 | Adenocarcinoma | II-III | DG | 4a | 1 | 0 | 1b | Body | 3 |  | No | No | 105 | 0 | Low | Low |
| H14 | Female | 47 | Signet_ring_cell_carcinoma |  | PG | 4a | 0 | 0 | 2b | Body | 2 | Infiltrative_ulcerative | No | No | 110 | 0 | Low | Low |
| A7 | Female | 62 | Adenocarcinoma | II | DG | 4a | 0 | 0 | 1a | Antrum | 3 |  | Yes | No | 115 | 0 | Low | Low |
| C13 | Male | 54 | Adenocarcinoma | II-III | DG | 4a | 2 | 0 | 3b | Antrum | 7 | Diffuse_infiltrative | No | No | 116 | 0 | Low | Low |
| F1 | Male | 45 | Adenocarcinoma | III | PG | 4a | 1 | 0 | 3a | Greater_curvature | 4 | Infiltrative_ulcerative | Yes | No | 120 | 0 | Low | Low |

**Supplementary table 3**

| **Antibodies** | **Source** | **Identifier** |
| --- | --- | --- |
| **APC/Cy7 anti-mouse CD8a Antibody** | **BioLegend** | **Cat. No. 100714** |
| **FITC anti-mouse CD8b Antibody** | **BioLegend** | **Cat. No. 126606** |
| **PerCP/Cyanine5.5 anti-mouse CD45 Antibody** | **BioLegend** | **Cat. No. 103132** |
| **PE Anti-Mouse IFN gamma** | **BioLegend** | **Cat. No. 505808** |
| **Pacific Blue^TM^ anti-human/mouse Granzyme B Antibody** | **BioLegend** | **Cat. No. 515408** |
| **FITC anti-mouse/human CD11b Antibody** | **BioLegend** | **Cat. No. 101205** |
| **APC anti-mouse F4/80 Antibody** | **BioLegend** | **Cat. No. 123116** |
| **PerCP anti-mouse Ly-6C Antibody** | **BioLegend** | **Cat. No. 128028** |
| **Anti-HADHA Antibody** | **Abcam** | **Ab203114** |
| **Anti-HADHA K406 la Antibody** | **PTMBIO** |  |
| **Anti-PHGDH Antibody** | **Abcam** | **Ab57030** |
| **Anti-STAT3 (phospho Y705) Antibody** | **Abcam** | **Ab267373** |
| **Anti-STAT3 Antibody** | **Abcam** | **Ab68153** |
| **Anti-RIG-I/DDX58 Antibody** | **Abcam** | **Ab180675** |
| **Anti-AMPK beta 1 Antibody** | **Abcam** | **Ab32112** |
| **Anti-HADHB Antibody** | **Abcam** | **Ab230667** |
| **Anti-SREBP2 Antibody** | **Abcam** | **Ab30682** |
| **Anti-TORC2 Antibody** | **Abcam** | **Ab109081** |
| **Acetyl-Histone H2A.Z (Lys4/Lys7)** | **CST** | **75336** |
| **Acetyl-Histone H2B (Lys5)** | **CST** | **12799** |
| **Acetyl-Histone H3 (Lys9)** | **CST** | **9649** |
| **Acetyl-Histone H3 (Lys27)** | **CST** | **8173** |
| **Acetyl-Histone H3 (Lys36)** | **CST** | **27683** |

**Supplementary Table 4**

| gene | Primers for ChiP-PCR or qRT-PCR |
| --- | --- |
| Human HMGCR#1 forward primer | 5’-TCGTAAGGCAGCAAACACGTA-3’ |
| Human HMGCR#1 reverse primer | 5’-CGAAAGCACGGAGCAGTCTT-3’ |
| Human HMGCR#2 forward primer | 5’-AAAGCTGGGCCCCAAAGA-3’ |
| Human HMGCR#2 reverse primer | 5’-GCTTGTTCCCGGCATTCC-3’ |
| Human HMGCR#3 forward primer | 5’-CGCGAGGAGGAGCAACTG-3’ |
| Human HMGCR#3 reverse primer | 5’-AGGAGCTCACATCCCCATTG-3’ |
| Human CRTC2#1 forward primer | 5’-CAGCCGACGTGGGGGTATTC-3’ |
| Human CRTC2#2 reverse primer | 5’-ATGGGTGGCTGAGAGGTGAA-3’ |
| Human SREBP2#1 forward primer | 5’-AGCTGGTCTGTGAAGTCAGC-3’ |
| Human SREBP2#2 reverse primer | 5’-AAGGAACTCTGCTGCCCATC-3’ |
| Human XBP1#1 forward primer | 5’-AAGTTCTGCTTCTGTCGGGG-3’ |
| Human XBP1#2 reverse primer | 5’-GGCTGGTAAGGAACTGGGTC-3’ |
| Human iNOS#1 forward primer | 5’-TCCAAGGTATCCTGGAGCGA-3’ |
| Human iNOS#2 reverse primer | 5’-CAGGGACGGGAACTCCTCTA-3’ |
